# Coherent Spin Dynamics of Electrons in CdSe Colloidal Nanoplatelets

**DOI:** 10.3390/nano13233077

**Published:** 2023-12-04

**Authors:** Sergey R. Meliakov, Vasilii V. Belykh, Ina V. Kalitukha, Aleksandr A. Golovatenko, Alessio Di Giacomo, Iwan Moreels, Anna V. Rodina, Dmitri R. Yakovlev

**Affiliations:** 1 P.N. Lebedev Physical Institute of the Russian Academy of Sciences, 119991 Moscow, Russia; 2 Experimentelle Physik 2, Technische Universität Dortmund, 44227 Dortmund, Germany; 3 Ioffe Institute, Russian Academy of Sciences, 194021 St. Petersburg, Russia; kalitukha@gmail.com (I.V.K.); sasha.pti@mail.ioffe.ru (A.A.G.);; 4 Department of Chemistry, Ghent University, 9000 Ghent, Belgium

**Keywords:** colloidal nanocrystals, CdSe nanoplatelets, time-resolved Faraday rotation, coherent spin dynamics, Landè factor, spin–flip Raman scattering

## Abstract

Coherent spin dynamics of electrons in CdSe colloidal nanoplatelets are investigated by time-resolved pump–probe Faraday rotation at room and cryogenic temperatures. We measure electron spin precession in a magnetic field and determine *g*-factors of 1.83 and 1.72 at low temperatures for nanoplatelets with a thickness of 3 and 4 monolayers, respectively. The dephasing time of spin precession $T_{2}^{*}$ amounts to a few nanoseconds and has a weak dependence on temperature, while the longitudinal spin relaxation time $T_{1}$ exceeds 10 ns even at room temperature. Observations of single and double electron spin–flips confirm that the nanoplatelets are negatively charged. The spin–flip Raman scattering technique reveals *g*-factor anisotropy by up to 10% in nanoplatelets with thicknesses of 3, 4, and 5 monolayers. In the ensemble with a random orientation of nanoplatelets, our theoretical analysis shows that the measured Larmor precession frequency corresponds to the in-plane electron *g*-factor. We conclude that the experimentally observed electron spin dephasing and its acceleration in the magnetic field are not provided by the electron *g*-factor anisotropy and can be related to the localization of the resident electrons and fluctuations of the localization potential.

## 1. Introduction

Colloidal chemical synthesis is a common method for fabricating semiconductor nanocrystals of various shapes and sizes. Colloidal CdSe nanoplatelets (NPLs) have thicknesses of several monolayers (MLs) and large lateral dimensions. They exhibit bright optical properties [1,2,3,4], such as high stability, large absorption cross sections [5], and narrow spectral lines [6], which are very attractive for their optoelectronics applications [7,8]. High optical gain and amplification of spontaneous emission with a low threshold have also been reported [9,10,11]. Even transistors can be fabricated based on NPLs due to their large lateral dimensions [12].

Colloidal NPLs are convenient model materials for studying the electronic, optical, and spin properties of two-dimensional (2D) systems. However, their spin properties are still poorly investigated and understood compared to epitaxially grown semiconductor quantum wells. Despite their similar thicknesses, their properties can be very different. Thus, knowledge of the spin properties gained from epitaxial nanostructures can be used for NPLs only after careful experimental and theoretical verification.

Excitons, which are bound states of an electron and a hole, are very prominent in optical spectra of CdSe NPLs. In bulk CdSe, the exciton binding energy is 15 meV [13,14,15]. However, it increased greatly in the CdSe NPLs and reaches hundreds of meV [16,17,18,19,20]. Photoluminescence (PL) spectrum of the CdSe NPLs typically consists of two lines separated by 10–30 meV depending on the NPL thickness. The low-energy line corresponds to a negatively charged exciton (trion), and the high-energy line is related to a neutral exciton [21,22,23]. The neutral exciton ground state is a doubly degenerate dark state, which is 3–6 meV lower in energy than the bright exciton state [24,25].

Spin studies of CdSe nanocrystals started more than 20 years ago [26,27,28] and still attract attention of researchers [29,30,31,32]. A very unusual effect has been found, i.e., the manifestation of surface spins, which strongly polarize exciton spins in CdSe NPLs at low temperatures [33]. Spin properties and electron *g*-factors have been studied by spin–flip Raman scattering (SFRS) techniques in CdSe/CdS core/shell NPLs with thick shells [34] and in bare-core CdSe NPLs [35]. Also, the optical alignment and optical orientation of excitons have recently been studied in CdSe/CdS core/shell NPLs [36].

A very powerful and informative experimental tool to study spin coherence and other spin properties of semiconductor nanostructures is the time-resolved pump-probe Faraday rotation (TRFR) technique [37]. It has been successfully used for colloidal II-VI nanocrystals in the form of quantum dots, both grown by wet chemistry in solution and synthesized in glass [38,39,40,41,42,43,44,45,46,47,48,49]. For NPLs there are only two of such studies, which are mostly focused on CdSe/CdS core/shell NPLs at room temperature [34,50]. In Ref. [50] the results for bare-core 4 ML CdSe NPLs were presented, whereas the main focus was shifted to CdSe/CdS core/shell NPLs. These experiments have been performed for NPLs in solution. In Ref. [34] CdSe/CdS core/shell NPLs with thick shells have been investigated. Thus, the experimental information on spin dynamics in CdSe NPLs is very limited, especially, on their temperature dependence. This motivates us to study systematically the spin dynamics of bare-core CdSe NPLs at wide temperature range.

In this paper, we present comprehensive studies of the coherent spin processes in bare-core CdSe NPLs. We measure coherent spin dynamics in 3 and 4 ML CdSe NPLs by means of TRFR in the temperature range from 5 K up to room temperature. To measure *g*-factor, inhomogeneous spin dephasing time $T_{2}^{*}$, and longitudinal spin relaxation time $T_{1}$ of electrons we use tilted magnetic fields. We attribute the observed spin beats to resident electrons with nanosecond-long spin dephasing time $T_{2}^{*}$ in the whole temperature range. Our experiments revealed long longitudinal spin relaxation with characteristic times $T_{1}$ exceeding 10 ns at room temperature. Such long spin relaxation times at a room temperatures are untypical for semiconductor structures and revealed for the first time in NPLs. We show that electron *g*-factor increases with decreasing NPL thickness and shows unexpected increase with temperature. The SFRS measurements reveal electron *g*-factor anisotropy and evidence the presence of at least two localized resident electrons in some NPLs by detecting the double electron spin-flip. We show theoretically why the anisotropy of the electron *g*-factor is not observed in TRFR studies. We analyze theoretically the origin of the electron spin inhomogeneous dephasing in the transverse magnetic field and show that it is only weakly contributed by the spread of NPL lateral sizes and electron *g*-factor anisotropy, even in the case of randomly oriented NPLs in the ensemble. We conclude that the inhomogeneous spin dephasing could be caused by the electron *g*-factor dispersion due to different electron localization conditions in the NPLs, and by presence of charged impurities near the NPLs surface resulting in Rashba-type spin-orbit coupling [51,52].

## 2. Materials and Methods

### 2.1. Samples

We studied CdSe NPLs with thicknesses of 3 ML, 4 ML, and 5 ML obtained by colloidal synthesis. In particular, 3 ML NPLs were prepared according to Di Giacomo et al. [4] using cadmium octanoate as the precursor; 4 ML NPLs were prepared according to Bertrand et al. [3] using cadmium acetate dihydrate to induce 2D growth, and 5 ML NPLs were prepared according to Ithurria et al. [2]. The NPLs had a rectangular shape with a width of 7±2 nm (7.8±2 for 5 ML) and lengths of 37±16 nm (3 ML), 29±4 nm (4 ML), and 30±2 nm (5 ML). For optical measurements, a concentrated solution of CdSe NPL was drop-cast on a glass or silicon substrate and dried. Some measurements on spin dynamics at room temperature were performed for NPLs in a solution.

### 2.2. Time-Resolved Faraday Rotation

The coherent spin dynamics are measured by the time-resolved pump–probe Faraday rotation (TRFR) technique. The laser system consisting of a Yb-KGW (Ytterbium doped potassium gadolinium tungsten) laser (PHAROS, Light Conversion, Ltd., Vilnius, Lithuania) integrated with a regenerative amplifier, combined with a narrow-band picosecond optical parametric amplifier (ps-OPA, ORPHEUS-PS Light Conversion Ltd.) generates picosecond pulses with a spectral width of about 1 nm and a repetition rate of 25 kHz (repetition period 40 μs). The laser photon energy is tunable in the spectral range of 0.47–3.88 eV (320–2600 nm). The laser beam is split into pump and probe beams. To create a controlled delay between the pump and probe, the pump beam is passed through a mechanical delay line with a retroreflector. The pump beam is modulated with an electro-optical modulator between $\sigma^{+}$ and $\sigma^{-}$ polarizations at a frequency of 26 kHz to eliminate background signal and nuclear spin effects. The probe beam is linearly polarized. The pump and probe average powers are 200 μW and 150 μW, respectively. The diameter of the pump and probe beam spots is about 100 μm. The Faraday rotation angle of the probe beam, which is directly proportional to the spin polarization in the sample, is measured using a Wollaston prism, a balanced photodetector, and a lock-in amplifier, synchronized with the electro-optical modulator.

For magneto-optical measurements, samples are placed in a helium flow cryostat. The sample temperature is set in a range from 5 K to 295 K. When measuring the temperature dependence of spin dynamics, a magnetic field generated by a permanent magnet is applied at an angle $\alpha =35^{\circ }$ to the normal of the substrate. To study the magnetic field dependence, the cryostat with the sample is placed between two poles of an electromagnet, providing Voigt magnetic fields of up to 430 mT.

### 2.3. Time-Resolved Differential Transmission

To study the dynamics of the differential transmission ΔT/T, we use a pump–probe technique similar to that described above with the same laser system. Here, the pump pulse is linearly polarized and amplitude-modulated at a frequency of 52 kHz. Photogenerated carriers in NPLs modify transmission, the dynamics of which are detected by a linearly polarized probe pulse measured by a photodetector and lock-in amplifier.

### 2.4. Time-Resolved Photoluminescence

To study the PL dynamics, we use a streak camera (with a time resolution of 5 ps) coupled with a 0.5 m spectrometer (with a spectral resolution of 1 nm). The time resolution of the whole system “streak camera and spectrometer” is about 10 ps for short-range (<2 ns) measurements with the SynchroScan streak camera module and is correspondingly reduced for long-range (>2 ns) measurements with the single sweep module. In these experiments, the samples are excited by the second harmonics of Ti:Sapphire laser pulses. The pulses, with a duration of 1 ps and a repetition rate of 76 MHz, are generated at a photon energy of 1.722 eV (720 nm) and frequency is doubled to 3.444 eV (360 nm).

### 2.5. Spin–Flip Raman Scattering

SFRS measurements are performed at a cryogenic temperature of 2 K for samples in contact with pumped liquid helium. A split-coil superconducting solenoid is used to apply magnetic fields up to 5 T in Voigt (perpendicular to the optical axis) and Faraday (parallel to the optical axis) geometries. The SFRS is measured in a backscattering geometry. For excitation, we use emission lines of an Ar-ion laser: 2.541 eV (488 nm), 2.497 eV (476.5 nm), and 2.471 eV (501.7 nm), a He-Cd laser: 2.808 eV (441.6 nm), and an Nd:YAG laser: 2.331 eV (532 nm). The laser power density on the sample surface is about 10 Wcm${}^{-2}$. The scattered light is analyzed by a Jobin-Yvon U1000 double monochromator equipped with a cooled GaAs photomultiplier connected to conventional photon counting electronics. To record a sufficiently strong SFRS signal and to suppress the laser stray light, spectral slit widths of 0.2 cm${}^{-1}$ (0.025 meV) are used. To characterize the polarization properties of the SFRS lines, conventional polarization optics are used, such as λ/4-plates and Glan–Thompson prisms.

### 2.6. Photoluminescence

At a temperature of 5 K, PL is detected with the experimental setup used for time-resolved PL measurements and is integrated over time. At T=2 K, the PL is measured with the setup used for the SFRS. The samples are excited by an Ar-ion laser at 2.410 eV (514.5 nm) in the case of 3 ML and 4 ML NPLs and by the solid-state laser with a photon energy of 3.062 eV (405 nm) in the case of 5 ML NPLs. The PL is detected by a Jobin-Yvon U1000 double monochromator equipped with a cooled GaAs photomultiplier.

## 3. Results

**Figure 1:**
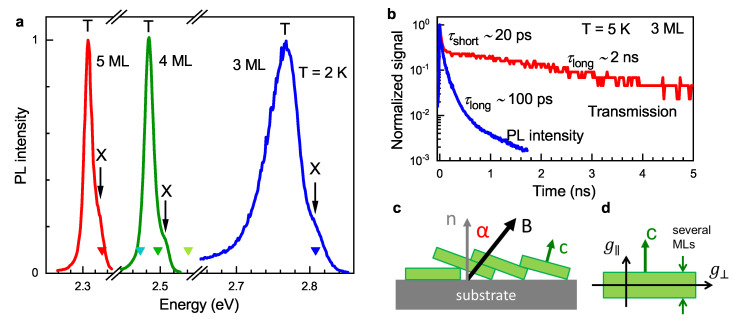
(**a**) Photoluminescence spectra of CdSe NPLs measured at T=2 K. The samples are excited by a continuous wave laser with a photon energy of 3.062 eV for 3 and 4 ML and 2.410 eV for 5 ML NPLs. Exciton (X) and trion (T) lines are marked. Triangles show the excitation energies used for the SFRS measurements. (**b**) Dynamics of time-resolved differential transmission (red) measured at 2.762 eV and PL (blue), excited at 3.444 eV, and integrated over the spectrum of 3 ML NPLs. Times of the short and long components, $\tau_{\mathrm{short}}$ and $\tau_{\mathrm{long}}$, are obtained by fitting experimental data with the multi-exponential decay function. (**c**) Schematics of NPLs on the substrate in a magnetic field. n is the normal to the substrate plane, c is the normal to the NPL plane (anisotropy axis), and α is an angle between the n and magnetic field B. (**d**) Schematics of *g*-factor anisotropy in NPL. $g_{\left|\right|}$ and $g_{⊥}$ correspond to the *g*-factor in the magnetic field directed along and perpendicular to c, respectively.

We studied CdSe NPLs with thicknesses of 3 ML, 4 ML, and 5 ML, which were synthesized by colloidal chemistry. Their PL spectra—measured at a temperature of T=2 K—are shown in Figure 1a. One can see that the emission spectrum shifts to higher energies with decreasing NPL width, which is due to the carrier quantum confinement. The spectral energies of PL agree well with the literature data [21,24]. All spectra have double-line structures, where the dominating low-energy line corresponds to the trion (negatively charged exciton) recombination, and the weak high-energy line is related to the exciton recombination [21,24]. The energy difference between line maxima corresponds to the trion-binding energy. It reaches about 40 meV in 3 ML NPLs and decreases to 20 meV in 5 ML NPLs, which is consistent with the values in Refs. [21,24]; see Figure S4, Supplementary Materials.

Figure 1b shows the population dynamics in 3 ML NPLs measured by time-resolved differential transmission and PL. The differential transmission is detected at a photon energy of 2.762 eV, while PL is integrated over the whole spectrum. Both dynamics are multi-exponential, with the characteristic time of the shortest component $\tau_{\mathrm{short}}\approx 20$ ps. At longer times, the differential transmission and PL decay with times $\tau_{\mathrm{long}}$ of 2 ns and 100 ps, respectively. For NPLs, at cryogenic temperatures, the trion and exciton emissions differ drastically in their recombination dynamics: trion has a single fast decay time of 90 ps, and exciton has a two-component decay corresponding to bright (10 ps) and dark (46 ns at T=4.2 K) excitons [21]. Thus, we assign the short PL component with 20 ps to bright excitons and the long one with 100 ps to trion recombination. The dark exciton is not visible in the PL dynamics, since at low temperatures it has a very long decay time and very low amplitude [21], which hinders its detection within the intensity dynamical range of the streak camera. The differential transmission reflects changes in exciton and trion populations, as well as the redistribution of exciton and trion oscillator strengths as a result of photocharging. Accordingly, the fast dynamics of 20 ps can correspond to the population of bright excitons (the effect that they bleach absorption) and the long dynamics of 2 ns or longer (as we are limited by a time range) are due to NPL photocharging (Supplementary Materials, Section S1). A comparison of the differential transmission measured at energies of 2.762 eV and 2.749 eV is presented in Figure S1, Supplementary Materials.

### 3.1. Coherent Spin Dynamics at T=5 K

To study the spin dynamics in CdSe NPLs, we use a time-resolved Faraday rotation technique. In these experiments, an external magnetic field B is applied in the Voigt geometry, being perpendicular to the optical axis *z* and the normal to the substrate plane n: B⊥n. It corresponds to angle $\alpha =90^{\circ }$ in Figure 1c. Note that individual NPLs in the studied ensembles can be oriented at various angles to the substrate normal, so that the normal to the NPL plane (anisotropy c-axis, c) is not necessarily parallel to n.

**Figure 2:**
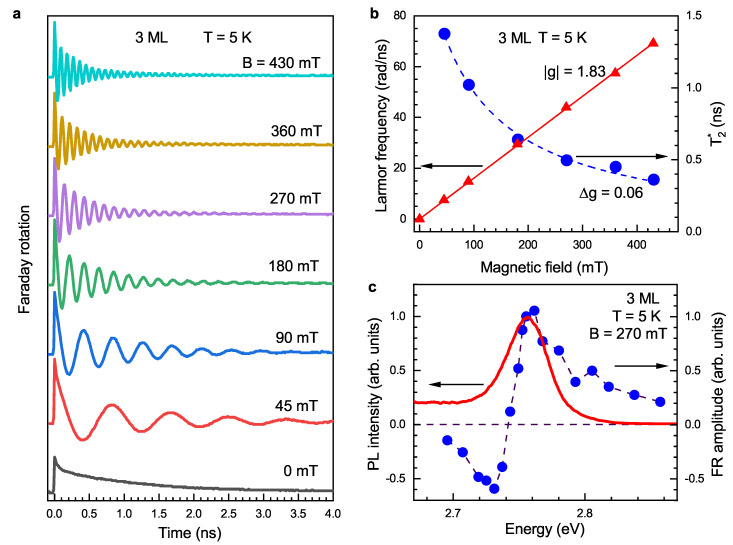
Spin dynamics in 3 ML CdSe NPLs at T=5 K. (**a**) TRFR measured at different Voigt magnetic fields. The photon spectral energy is 2.755 eV. (**b**) The magnetic field dependence of Larmor precession frequency (red triangles) and inhomogeneous transverse spin dephasing time $T_{2}^{*}$ (blue circles). The Larmor precession frequency increases linearly with the magnetic field and its slope fitted with Equation (1) (solid red line) gives |g|=1.83. The dashed line shows the fit of the data for $T_{2}^{*}\left(B\right)$ by Equation (3) with Δg=0.06. (**c**) The PL spectrum (red curve) and spectral dependence of FR amplitude (blue circles) at B=270 mT. PL is excited by the picosecond laser pulsed with a photon energy of 3.444 eV.

Figure 2a shows that the Faraday rotation (FR) dynamics in 3 ML NPLs measured in magnetic fields varied from 0 up to 430 mT. At a zero magnetic field, the nonoscillatory decay with a time of 1 ns is observed. In nonzero magnetic fields, the dynamics have damped oscillations, which reflect Larmor precession and dephasing of the spin polarization, S(t), created by pump pulses. The Larmor frequency of the spin precession, $\omega_{L}$, is determined by the Landé *g*-factor and scaled with the magnetic field strength according to

(1) $$\omega_{L}=\left|g\right|\mu_{B}B/\hbar \;.$$

Here $\mu_{B}$ is the Bohr magneton. The oscillation damping is described by the spin dephasing time $T_{2}^{*}$, which characterizes the inhomogeneity of the spin ensemble. The Faraday rotation dynamics can be approximated by

(2) $$S\left(t\right)=S_{0}\exp\left(-t/T_{2}^{*}\right)\cos\left(\omega_{L}t\right),$$

where $S_{0}$ is the initial spin polarization photogenerated along the optical axis.

Fitting the experimental data with Equation (2) allows us to evaluate the Larmor precession frequency and the spin dephasing time. Their magnetic field dependences are shown in Figure 2b. The Larmor precession frequency is proportional to *B* according to Equation (1), giving |g|=1.83 for 3 ML NPLs. We show below that this *g*-factor can be assigned to electrons. The spin dephasing time $T_{2}^{*}$ decreases with the growing magnetic field, which is typical for inhomogeneous ensembles with a finite spread of the *g*-factor distribution Δg. This dependence can be described by the equation given in Ref. [37]:

(3) $$\frac{1}{T_{2}^{*}\left(B\right)}\approx \frac{1}{T_{2}^{*}\left(0\right)}+\frac{\Delta g\mu_{B}B}{\hbar }.$$

Here, $T_{2}^{*}\left(0\right)$ is the spin dephasing time at the zero magnetic field. The second term is responsible for inhomogeneous dephasing in the magnetic field. Fitting the experimental data in Figure 2b with Equation (3) yields Δg=0.06 for 3 ML NPLs at T=5 K. Room temperature measurements give Δg=0.08 for 3 ML and 0.17 for 4 ML NPLs (Supplementary Materials, Section S3). Below, we theoretically analyze and discuss the possible origins of the *g*-factor spread, particularly the role of *g*-factor anisotropy and the random orientation of NPLs.

The spectral dependence of the FR amplitude measured at B=270 mT is shown in Figure 2c. It has a typical dispersion-like shape in the vicinity of the trion resonance and reaches a maximum near the PL maximum. The FR amplitude dispersion is in agreement with the theoretical analysis of Ref. [53], where the change in the amplitude sign at the trion resonance was explained. The *g*-factor has weak spectral dependence (Figure S2b, Supplementary Materials). The spin dephasing time $T_{2}^{*}$ is 0.3 ns at higher energies and increases to 0.6 ns at lower energies, which is presented and discussed in detail in the Supplementary Materials, Section S2.

### 3.2. Temperature Dependence of Spin Dynamics

In order to measure the temperature dependences of both transverse and longitudinal components of spin polarization and evaluate the corresponding transverse and longitudinal spin relaxation times, we use the tilted magnetic field geometry. The magnetic field B=50 mT is applied at an angle $\alpha =35^{\circ }$ to the substrate normal n, while the light is directed along n (Figure 1c). In such a case, the spin dynamics are described as follows:

(4) $$S\left(t\right)=S_{⊥}\sin\alpha \cos\left(\omega_{L}t\right)\exp\left(-t/T_{2}^{*}\right)+S_{∥}\cos\alpha \exp(-t/T_{1}).$$

Here, $S_{⊥}=S_{0}\sin\alpha$ and $S_{∥}=S_{0}\cos\alpha$ are the transverse and longitudinal components of the spin polarization with respect to the magnetic field, respectively, created by the pump pulses. The first term in Equation (4) is similar to Equation (2), while the second term describes the longitudinal spin relaxation with time $T_{1}$. In general, the longitudinal decay can be multi-exponential. Equation (4) does not take into account the anisotropy of the electron *g*-factor in the NPL. However, its account would not significantly modify the values of the spin dephasing times (see Figure S11).

**Figure 3:**
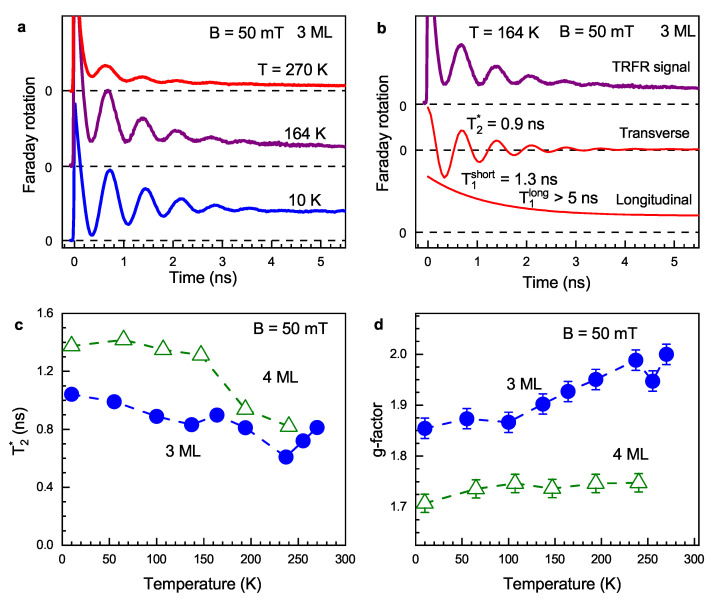
Temperature dependence of the spin dynamics in CdSe NPLs. Magnetic field of 50 mT is tilted by $\alpha =35^{\circ }$ with respect to the substrate normal. (**a**) FR dynamics at various temperatures in 3 ML NPLs. (**b**) FR dynamics in 3 ML NPLs at T=164 K (upper graph). The middle and lower graphs show transverse and longitudinal components of experimentally measured spin dynamics. The components are distinguished by fitting with Equation (4). (**c**) Temperature dependence of $T_{2}^{*}$ time for 3 ML (blue circles) and 4 ML (open green triangles) NPLs. (**d**) Temperature dependence of *g*-factor for 3 ML (blue circles) and 4 ML (open green triangles) NPLs.

FR dynamics measured at different temperatures in 3 ML NPLs are shown in Figure 3a. Here, we present data up to 270 K, while similar results at room temperature are presented in the Supplementary Materials (Sections S3 and S4). Even at room temperature, we observe long transverse and longitudinal spin dynamics. Fitting with Equation (4) allows us to separate them and evaluate *g*-factors and spin relaxation times $T_{1}$ and $T_{2}^{*}$. An example of such an evaluation for the spin dynamics at T=164 K is presented in Figure 3b. The transverse component yields a *g*-factor of 1.92 and $T_{2}^{*}=0.9$ ns. The longitudinal relaxation has two components, short and long, manifested as decay and constant levels, respectively, in the lower part of Figure 3b. $T_{1}^{\mathrm{short}}$ is about 1–2 ns, and it varies slightly with temperature (Table S1, Supplementary Materials). The characteristic time corresponding to the long component $T_{1}^{\mathrm{long}}$, even at room temperature, is much longer than the maximum delay of 6 ns in our experiment. Therefore, we conclude that $T_{1}^{\mathrm{long}}\gg 10$ ns.

Figure 3c shows temperature dependences of $T_{2}^{*}$ in 3 ML and 4 ML NPLs. At T=10 K, the times are $T_{2}^{*}=1.0$ ns for 3 ML NPLs and 1.4 ns for 4 ML NPLs. The time decreases with temperature down to values of about 0.7 ns at T=250 K. This result is unusual for semiconductor structures, where commonly, phonon scattering drastically accelerates spin relaxation at elevated temperatures. Figure 3d presents temperature dependences of *g*-factors. For 4 ML NPLs, it is approximately constant at the level of 1.72, but for 3 ML NPLs, the *g*-factor increases by 10% with the temperature ranging from 1.85 to 2.0. This result contradicts the expected behavior of semiconductor systems, where, according to the Roth–Lax–Zwerdling equation [54], the decrease in band gap energy with temperature should lead to a decrease in the electron *g*-factor.

### 3.3. Electron *g*-Factor Anisotropy Measured by Spin–Flip Raman Scattering

We measure the electron *g*-factor and its anisotropy in 3, 4, and 5 ML NPLs by means of spin–flip Raman scattering. The experiments are performed at the cryogenic temperature of 2 K, in magnetic fields applied in Voigt (B⊥z‖n), Faraday (B‖z‖n), or tilted geometries. Figure 1c illustrates the experimental geometries, where α is the angle between the magnetic field B and the normal to the substrate surface n. Polarization properties of SFRS are measured in two configurations: linear cross-polarization (V-excitation and H-detection) and linear co-polarization (H-excitation and H-detection). Notations H (horizontal) and V (vertical) are used for the parallel and perpendicular orientations of the photon electrical vector, with respect to the magnetic field direction, which is parallel to the horizontal direction.

**Figure 4:**
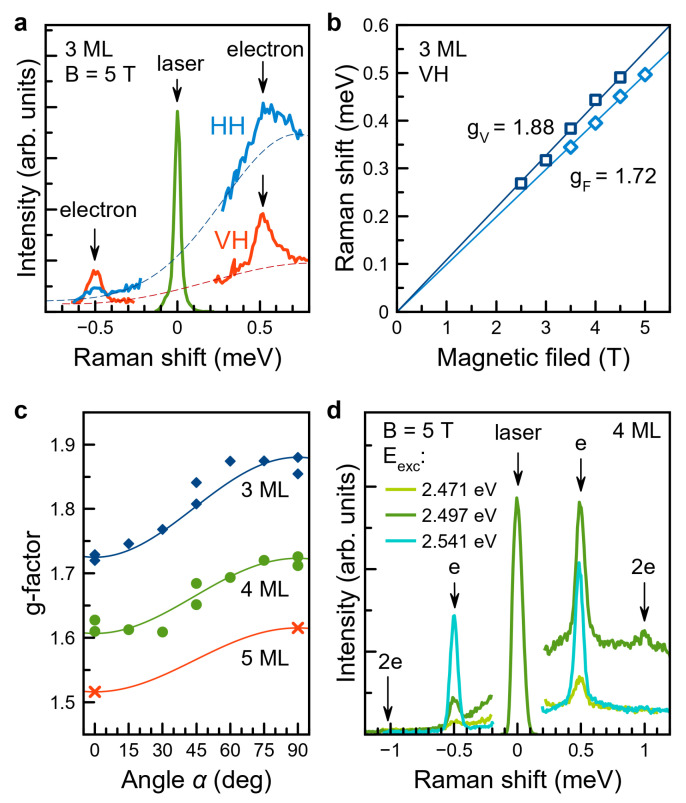
Spin–flip Raman scattering in CdSe NPLs. (**a**) SFRS spectra of 3 ML CdSe NPLs measured under resonant excitation of exciton at 2.808 eV (see Figure 1a) in cross- (red) and co- (blue) linear polarizations. Magnetic field B=5 T is applied in Faraday geometry, T=2 K. Dashed lines represent the resonant PL background. (**b**) Magnetic field dependence of the Raman shift of the electron spin–flip line measured in the Voigt (dark blue squares) and Faraday (light blue diamonds) geometries for excitation at 2.808 eV of 3 ML NPLs. The shifts are evaluated from the anti-Stokes area of VH-polarized spectra. Solid lines are linear fits of the data. (**c**) Anisotropy of electron *g*-factor in NPLs of different thicknesses: 3 ML (blue diamonds), 4 ML (green circles), and 5 ML (red crosses). (**d**) SFRS spectra of 4 ML CdSe NPLs measured at different excitation energies. Faraday magnetic field B=5 T, T=2 K. Here, “e” stands for electron spin–flip and “2e” for double electron spin–flip.

As an example of polarized SFRS, we present the data for 3 ML NPLs. Figure 4a shows SFRS spectra in co- and cross-polarizations measured under resonant excitation of the exciton at $E_{\mathrm{exc}}=2.808$ eV in the Faraday magnetic field B=5 T. Stokes (positive Raman shifts) and anti-Stokes (negative Raman shifts) areas of the spectra exhibit a broad background of resonant PLs (approximately shown by thin dashed curves) and lines separated by the energy at around ±0.5 meV from the laser line. These lines are attributed to the electron spin–flip Raman scattering process. Since the Stokes area of the spectrum, to a larger extent, is influenced by resonant PL, the polarization properties there are not as clear. In contrast, in the anti-Stokes area, where resonant PL is negligible, the electron spin–flip line is four times more intense in cross-polarizations (V-excitation and H-detection) than in co-polarizations (V-excitation and V-detection). The observation of spin-flip lines under resonant excitation of the exciton in cross-polarizations indicates that the mechanism of electron spin–flip is related to the interaction between a resident electron and a photoexcited exciton, as suggested in Ref. [35] for similar bare-core CdSe NPLs. The presence of a resident electron in an NPL means that this NPL is negatively charged.

The magnetic field dependence of the Raman shift is shown in Figure 4b for two geometries: Faraday ($\alpha =0^{\circ }$, light blue diamonds) and Voigt ($\alpha =90^{\circ }$, dark blue squares). From the linear fit of these dependences, one can determine the *g*-factor via electron Zeeman splitting $\Delta =\mu_{B}\left|g\right|B$. For 3 ML NPLs, this fit yields Voigt and Faraday *g*-factors $g_{V}=1.88$ and $g_{F}=1.72$, respectively. The typical accuracy for *g*-factor values in SFRS experiments is better than ±0.1. The measured values of the *g*-factor correspond to the electron *g*-factor calculated in Ref. [55]. The difference between $g_{V}$ and $g_{F}$ originates from the anisotropy of the electron *g*-factor in a CdSe NPL due to its natural low symmetry (Figure 1d): the component of the *g*-factor tensor $g_{\|}$ (parallel to the normal to NPL plane c) differs from $g_{⊥}$ (perpendicular to c). In a completely randomly oriented ensemble, this difference is not observable. Thus, the observation of different *g*-factors in Voigt and Faraday geometries reveals the preferential orientation of NPLs on the substrate.

Polarization and angular properties of SFRS are analogous to what we reported for CdSe NPLs in Ref. [35]. In this work and Ref. [56] it was theoretically shown that the signal from a single electron spin flip in Voigt geometry comes from NPLs oriented horizontally on the substrate so that $g_{V}$ represents $g_{⊥}$. In turn, in Faraday geometry this signal should not be observed for NPLs oriented either strictly vertical (standing on the substrate, c⊥n) or strictly horizontal (laying on the substrate, c‖n). Nevertheless, it is clearly visible in the experiment (see Figure 4a and S5). Theoretical calculations predict a clearly distinguishable signal in Faraday geometry in two cases: if the NPLs are slightly tilted from 1) vertical and 2) horizontal orientation. In the first case, the observed *g*-factor $g_{F}$ must be equal to $g_{V}$ and they both correspond to $g_{⊥}$ of an individual NPL. In the second case, corresponding to our experimental data, $g_{F}$ differs from $g_{V}$ and approximately represents $g_{\|}$ of individual NPL. Even for such off-plane NPLs (schematics of such ensemble is given in Figure 1c) the theory predicts rather strict selection rules: line, corresponding to the single electron spin flip should be observed in cross linear polarizations in the Faraday geometry. This corresponds to our experimental findings, see Figure 4a and Figure S5.

**Figure 5:**
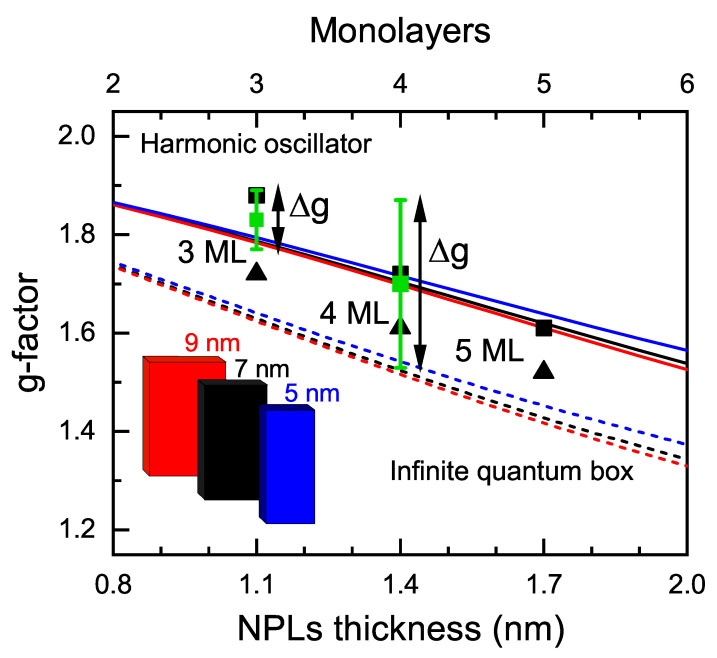
Calculated dependence of the electron *g*-factor on the NPL thickness for the CdSe NPL width 9 nm (red), 7 nm (black), 5 nm (blue), and infinite length. Dashed lines show the results of the calculations for infinite quantum box potential. Solid lines show the results of the calculation for the harmonic oscillator potential with an oscillator length of $L_{\mathrm{osc}}=L/4$ providing 95% probability of finding the electron inside the NPL. Black symbols show the SFRS data points for three NPL thicknesses. Squares and triangles correspond to Voigt and Faraday geometries, respectively. Green squares show *g*-factors determined from TRFR at low temperatures. The error bar for the 3 ML sample shows the Δg spread determined from the dependence of $T_{2}^{*}$ on the magnetic field at T=5 K. The error bar for the 4 ML sample shows the Δg spread determined from the dependence of $T_{2}^{*}$ on the magnetic field at T=295 K.

SFRS spectra, containing electron spin-flip line with the same polarization properties, were also observed for 4 and 5 ML NPLs. Thus, in all samples we observe spin flip of the resident electron interacting with the photogenerated exciton [35]. Additional data on SFRS and its polarization properties in 4 ML NPLs are given in Supplementary Materials, Section S6. Data on electron *g*-factor and its anisotropy in 3, 4 and 5 ML NPLs are summarized in Figure 4c. NPLs of all three thicknesses demonstrate *g*-factor angular dependence. Solid lines here are given by $g\left(\alpha \right)=\sqrt{g_{F}^{2}\cos^{2}\alpha +g_{V}^{2}\sin^{2}\alpha }$, with *g*-factor values $g_{F}$ and $g_{V}$, summarized in Table 1 and Figure 5. The anisotropy in all three samples is due to NPLs favorable orientation on the substrate (in-plane and slightly off-plane NPLs). As it was mentioned, on the basis of the theory from Refs. [35,56], we can assign experimentally measured $g_{F}$ and $g_{V}$ to intrinsic NPL *g*-factor tensor components parallel and perpendicular to the NPL *c*-axis, $g_{\|}$ and $g_{⊥}$, respectively. Figure 4c shows that in general, the *g*-factor decreases with increasing of NPLs thickness in agreement with quantum confinement reduction and theoretical calculations, see Figure 5 and Supplementary Materials, Section S7.

The spectra of spin–flip Raman scattering on electrons in 4 ML NPLs are shown in Figure 4d. Here, we use excitation energies, shown by triangles in Figure 1a: excitation in trion, in exciton, and above exciton. Under resonant exciton excitation (the dark green spectrum in Figure 4d), we observe electron spin–flip lines at shifts of ±0.5 meV, as well as double electron spin–flip lines (2e) at ±1 meV. The latter corresponds to the mechanism where the photogenerated exciton interacts with two resident electrons, mediating their spin–flips. The observation of the double electron spin–flip means that some NPLs are doubly negatively charged, and the photoexcited exciton does not form a singlet trion state with either of them [56]. The Raman shift of the double electron spin–flip matches the doubled Raman shift of the single electron spin–flip. This means that the two resident electrons involved in the process do not interact with each other but only with the exciton. The absence of such interaction means that the resident electrons are localized sufficiently distant from each other in the NPL. The relative intensity of the double electron spin–flip is larger under resonant excitation of the exciton, supporting the mechanism of resident electron spin–flip via the photogenerated exciton.

Thus, using the SFRS technique, we find that the studied CdSe NPLs are negatively charged, and some of them are (at least) doubly negatively charged with resident localized electrons. We measure the electron *g*-factor and its anisotropy in NPLs of different thicknesses.

## 4. Discussion

We studied the electron spin coherence in CdSe NPLs of different thicknesses by means of time-resolved Faraday rotation. The fact that we address electron spins deserves detailed explanations. First, we note that the dominant line in the PL spectra corresponds to negatively charged excitons (trions), as identified by several experimental approaches in Ref. [21]. Second, the measured values of the *g*-factor correspond to the electron *g*-factor recently calculated in Ref. [55], while the calculated hole *g*-factor is close to zero. Thus, we can safely conclude that electron spin precession is observed in our experiments.

The values of the electron *g*-factors measured at low temperatures are shown in Figure 5, along with the results of the theoretical calculations, which take into account the thickness and lateral sizes of NPLs (details of the calculation are given in the Supplementary Materials, Section S7). The *g*-factor increases with decreasing NPL thickness from 1.6 for 5 ML to 1.8 for 3 ML, in agreement with theoretical calculations. Note that in bulk CdSe with zinc blende crystal structure, the electron *g*-factor is positive and equal to 0.42 [57]. The decrease in NPL thickness increases the quantum confinement energy, and the electron *g*-factor is increased according to the Roth–Lax–Zwerdling equation [54].

As shown in Figure 5, the harmonic oscillator model gives better agreement (compared to the infinite quantum box model) of the calculated *g*-factors measured in the TRFR and SFRS experiments. Therefore, we can say that NPLs are charged with electrons which are localized. The resident electron localization also follows from the observation of the double equidistant spin-flip lines in SFRS experiment.

The TRFR experiments show that dephasing time of the electron spin precession $T_{2}^{*}$ is about 1 ns and weakly depends on temperature, while the time of the longitudinal spin relaxation $T_{1}$ is longer than 10 ns at both helium and room temperatures. Such long spin relaxation times correspond to long-lived electrons remaining in NPLs as a result of photocharging, which is revealed in measurements of differential transmission dynamics (Figure 1b). Spin dephasing times of the order of a nanosecond at room temperature were reported for CdSe and CdS quantum dots [38,39,40,41,42,43,44]. However, we are not aware of the works reporting long decay of the longitudinal spin component at room temperature. Note also, that in GaAs-based bulk structures, quantum wells and quantum dots spin relaxation times can be in nanosecond or even microsecond range at liquid helium temperature, but they are dramatically shortened when temperature is increased by several tens of Kelvin and fall in few picoseconds range at room temperature.The mechanisms of spin dephasing and relaxation in the studied NPLs apart from the spread of *g*-factors need further investigation. One of the sources of the spin dephasing may be the spin-orbit coupling induced by charge impurities on NPL surface [51,52].

The magnetic field dependence of the spin coherence time $T_{2}^{*}$, which, for 3 ML NPLs, is shown in Figure 2b, is proportional to 1/B. This is typical for the mechanism related to the *g*-factor spread Δg, which we evaluate from these measurements as 0.08 and 0.17 for 3 and 4 ML NPLs, respectively, at room temperature, and Δg=0.06 for 3 ML NPLs at T=5 K. Let us discuss the possible origin of the observed Δg. Figure 5 shows that these Δg values are larger than the fluctuations of the *g*-factor caused by the fluctuations of the NPL lateral sizes. We suggest two possible sources of Δg in the studied NPLs: (1) anisotropy of the electron *g*-factor revealed by the SFRS; and (2) localization of the resident electron at NPL surfaces and/or edges and fluctuations of the localization conditions.

Observation of the *g*-factor anisotropy in SFRS is possible for ensembles where the preferable orientation of the NPLs is close to horizontally lying and slightly tilted [35,56]. Theoretical consideration of the effect of the observed *g*-factor anisotropy and different orientations of the NPLs in the ensemble on the electron dephasing in TRFR experiments in the Voigt geometry is presented in the Supplementary Materials, Section S8. We show that the spin dephasing time $T_{2}^{*}$ related to this effect should be in the nanosecond range even at B=450 mT (Figure S10, Supplementary Materials). Thus, the experimentally observed electron spin dephasing and its acceleration in the magnetic field are not provided by the electron *g*-factor anisotropy. The electron *g*-factor measured by TRFR corresponds to the transverse electron *g*-factor $g_{⊥}$. Therefore, we suggest that the measured Δg values are provided by the localization of the resident electrons in the NPL and the fluctuations of the localization potential.

Surprisingly, the electron *g*-factor in CdSe NPLs increases with temperature (Figure 3d). This is in contrast to the behavior expected from the Roth–Lax–Zwerdling equation [54] for II-VI and III-V semiconductors with a zinc blend lattice. A similar effect was observed for the temperature dependence of the *g*-factor in bulk GaAs and CdTe [58,59,60,61], where the *g*-factor increase with temperature was supposed to be a result of band parameters renormalization. However, in our case, it can also be contributed to by a change in the localization conditions.

## 5. Conclusions

We studied the spin properties of electrons confined in CdSe nanoplatelets with thicknesses of 3, 4, and 5 monolayers by means of time-resolved Faraday rotation and spin–flip Raman scattering. We observe coherent spin precession of electrons in a temperature range from 5 K up to 295 K and measure the electron *g*-factor, which corresponds to the transverse *g*-factor $g_{⊥}$ determined from spin–flip Raman scattering studies. We show theoretically that spin precession detected in TRFR experiments originates from NPLs lying on a substrate along the magnetic field and perpendicular to the laser beam (and slightly inclined), while NPLs “standing” on a substrate are invisible in TRFR experiments. The electron spin dephasing time of the order of a nanosecond and longitudinal spin relaxation time exceeding 10 ns at room temperature are found. Observations of double-electron spin–flip and its model consideration allow us to ascribe the observed coherent spin dynamics to localized electrons. Our theoretical analysis shows that the measured acceleration of electron spin dephasing in magnetic fields is provided not by the electron *g*-factor anisotropy or by the dispersion of electron *g*-factors due to the spread of NPLs’ lateral sizes, but by different localization conditions of resident electrons. This work demonstrates the possibility of the optical orientation of resident spins and their unusually long relaxation times in CdSe NPLs at room temperature. It opens new avenues for the fundamental investigation of underlying spin relaxation mechanisms in this system and its use in spintronics applications.

## Figures and Tables

**Table 1:** *g*-factor anisotropy in CdSe NPLs revealed by SFRS. T=2 K.

Sample	$g_{F}\approx g_{\|}$	$g_{V}=g_{⊥}$
3 ML	1.72	1.88
4 ML	1.61	1.72
5 ML	1.52	1.61

## Data Availability

The data presented in this study are available upon request from the corresponding author.

## Supplementary Materials

The following supporting information can be downloaded at https://www.mdpi.com/article/10.3390/nano13233077/s1, Section S1: Dynamics of photoluminescence and differential transmission at T=5 K and 295 K; Section S2: Spectral dependences of spin parameters in 3 ML NPLs; Section S3: The magnetic field dependence of the spin dephasing time at room temperature; Section S4: Longitudinal spin relaxation times $T_{1}$; Section S5: Trion binding energies; Section S6: Spin-flip Raman scattering in 4 ML NPLs; Section S7: Calculation of the electron *g*-factor dispersion caused by the dispersion of NPL lateral sizes; Section S8: Theory of TRFR in CdSe NPLs. Refs. [16,21,34,35,44,45,50,53,55,62,63,64,65,66,67,68] are cited in the supplementary materials.

## References

[1] Ithurria S., Dubertret B. (2008). Quasi 2D colloidal CdSe platelets with thicknesses controlled at the atomic level. J. Am. Chem. Soc..

[2] Ithurria S., Tessier M.D., Mahler B., Lobo R.P.S.M., Dubertret B., Efros A.L. (2011). Colloidal nanoplatelets with two-dimensional electronic structure. Nature Mater..

[3] Bertrand G.H.V., Polovitsyn A., Christodoulou S., Khan A.H., Moreels I. (2016). Shape control of zincblende CdSe nanoplatelets. Chem. Commun..

[4] Di Giacomo A., Rodà C., Khan A.H., Moreels I. (2020). Colloidal synthesis of laterally confined blue-emitting 3.5 monolayer CdSe nanoplatelets. Chem. Mater..

[5] Khan A.H., Bertrand G.H.V., Teitelboim A., Sekhar M.C., Polovitsyn A., Brescia R., Planelles J., Climente J.I., Oron D., Moreels I. (2020). CdSe/CdS/CdTe core/barrier/crown nanoplatelets: Synthesis, optoelectronic properties, and multiphoton fluorescence upconversion. ACS Nano.

[6] Tessier M.D., Javaux C., Maksimovic I., Loriette V., Dubertret B. (2012). Spectroscopy of single CdSe nanoplatelets. ACS Nano.

[7] Fan F., Kanjanaboos P., Saravanapavanantham M., Beauregard E., Ingram G., Yassitepe E., Adachi M.M., Voznyy O., Johnston A.K., Walters G. (2015). Colloidal CdSe_1-x_S_x_ nanoplatelets with narrow and continuously-tunable electroluminescence. Nano Lett..

[8] Chen Z., Nadal B., Mahler B., Aubin H., Dubertret B. (2014). Quasi 2D colloidal semiconductor nanoplatelets for narrow electroluminescence. Adv. Funct. Mater..

[9] Guzelturk B., Kelestemur Y., Olutas M., Delikanli S., Demir H.V. (2014). Amplified spontaneous emission and lasing in colloidal nanoplatelets. ACS Nano.

[10] She C., Fedin I., Dolzhnikov D.S., Demortiere A., Schaller R.D., Pelton M., Talapin D.V. (2014). Low-threshold stimulated emission using colloidal quantum wells. Nano Lett..

[11] Grim J.Q., Christodoulou S., Di Stasio F., Krahne R., Cingolani R., Manna L., Moreels I. (2014). Continuous-wave biexciton lasing at room temperature using solution-processed quantum wells. Nat. Nanotechnol..

[12] Jana S., Martins R., Fortunato E. (2022). Stacking-dependent electrical transport in a colloidal CdSe nanoplatelet thin-film transistor. Nano Lett..

[13] Segall B., Marple D.T.F., Aven M., Prener J.S. (1967). Physics and Chemistry of II–VI Compounds.

[14] Shionoya S., Pilkuhn M.H. (1974). Proceedings of the Twelfth International Conference on the Physics of Semiconductors: July 15–19, 1974 Stuttgart, Volume 12.

[15] Voigt J., Spiegelberg F., Senoner M. (1979). Band parameters of CdS and CdSe single crystals determined from optical exciton spectra. Phys. Status Solidi B.

[16] Shornikova E.V., Yakovlev D.R., Gippius N.N., Qiang G., Dubertret B., Khan A.H., Giacomo A.D., Moreels I., Bayer M. (2021). Exciton binding energy in CdSe nanoplatelets measured by one- and two-photon absorption. Nano Lett..

[17] Zelewski S.J., Nawrot K.C., Zak A., Gladysiewicz M., Nyk M., Kudrawiec R. (2019). Exciton binding energy of two-dimensional highly luminescent colloidal nanostructures determined from combined optical and photoacoustic spectroscopies. J. Phys. Chem. Lett..

[18] Ji B., Rabani E., Efros A.L., Vaxenburg R., Ashkenazi O., Azulay D., Banin U., Millo O. (2020). Dielectric confinement and excitonic effects in two-dimensional nanoplatelets. ACS Nano.

[19] Benchamekh R., Gippius N.A., Even J., Nestoklon M.O., Jancu J.-M., Ithurria S., Dubertret B., Efros A.L., Voisin P. (2014). Tight-binding calculations of image-charge effects in colloidal nanoscale platelets of CdSe. Phys. Rev. B.

[20] Scott R., Achtstein A.W., Prudnikau A.V., Antanovich A., Siebbeles L.D.A., Artemyev M., Woggon U. (2016). Time-resolved Stark spectroscopy in CdSe nanoplatelets: Exciton binding energy, polarizability, and field-dependent radiative rates. Nano Lett..

[21] Shornikova E.V., Yakovlev D.R., Biadala L., Crooker S.A., Belykh V.V., Kochiev M.V., Kuntzmann A., Nasilowski M., Dubertret B., Bayer M. (2020). Negatively charged excitons in CdSe nanoplatelets. Nano Lett..

[22] Vong A.F., Irgen-Gioro S., Wu Y., Weiss E.A. (2021). Origin of low temperature trion emission in CdSe nanoplatelets. Nano Lett..

[23] Antolinez F.V., Rabouw F.T., Rossinelli A.A., Keitel R.C., Cocina A., Becker M.A., Norris D.J. (2020). Trion emission dominates the low-temperature photoluminescence of CdSe nanoplatelets. Nano Lett..

[24] Shornikova E.V., Biadala L., Yakovlev D.R., Sapega V.F., Kusrayev Y.G., Mitioglu A.A., Ballottin M.V., Christianen P.C.M., Belykh V.V., Kochiev M.V. (2018). Addressing the exciton fine structure in colloidal nanocrystals: The case of CdSe nanoplatelets. Nanoscale.

[25] Biadala L., Liu F., Tessier M.D., Yakovlev D.R., Dubertret B., Bayer M. (2014). Recombination dynamics of band edge excitons in quasi-two dimensional CdSe nanoplatelets. Nano Lett..

[26] Kuno M., Nirmal M., Bawendi M.G., Efros A.L., Rosen M. (1998). Magnetic circular dichroism study of CdSe quantum dots. J. Chem. Phys..

[27] Gupta J.A., Awschalom D.D., Peng X., Alivisatos A.P. (1999). Spin coherence in semiconductor quantum dots. Phys. Rev. B.

[28] Johnston-Halperin E., Awschalom D.D., Crooker S.A., Efros A.L., Rosen M., Peng X., Alivisatos A.P. (2001). Spin spectroscopy of dark excitons in CdSe quantum dots to 60 T. Phys. Rev. B.

[29] Martin P.I., Panuganti S., Portner J.C., Watkins N.E., Kanatzidis M.G., Talapin D.V., Schaller R.D. (2023). Excitonic spin-coherence lifetimes in CdSe nanoplatelets increase significantly with core/shell morphology. Nano Lett..

[30] Flor B., Zeman C.J., Ma X., Schatz G.C. (2023). Wavelength-Dependent Spin Excitation with Circularly Polarized Light in CdSe Nanoplatelets. J. Phys. Chem. C.

[31] Jiang M., Zhang Y., Hu R., Men Y., Cheng L., Liang P., Jia T., Sun Z., Feng D. (2023). Methods for obtaining one single Larmor frequency, either *ν*1 or *ν*2, in the coherent spin dynamics of colloidal quantum dots. Nanomaterials.

[32] Jiang M., Men Y., Zhang Y., Cheng L., Wang Y., Jia T., Sun Z., Feng D. (2023). Anomalous laser-fluence dependence of electron spin excitation in CdS colloidal quantum dots: Surface effects. J. Phys. Chem. Lett..

[33] Shornikova E.V., Golovatenko A.A., Yakovlev D.R., Rodina A.V., Biadala L., Qiang G., Kuntzmann A., Nasilowski M., Dubertret B., Polovitsyn A. (2020). Surface spin magnetism controls the polarized exciton emission from CdSe nanoplatelets. Nat. Nanotechnol..

[34] Shornikova E.V., Biadala L., Yakovlev D.R., Feng D.H., Sapega V.F., Flipo N., Golovatenko A.A., Semina M.A., Rodina A.V., Mitioglu A.A. (2018). Electron and hole *g*-factors and spin dynamics of negatively charged excitons in CdSe/CdS colloidal nanoplatelets with thick shells. Nano Lett..

[35] Kudlacik D., Sapega V.F., Yakovlev D.R., Kalitukha I.V., Shornikova E.V., Rodina A.V., Ivchenko E.L., Dimitriev G.S., Nasilowski M., Dubertret B. (2020). Single and double electron spin-flip Raman scattering in CdSe colloidal nanoplatelets. Nano Lett..

[36] Smirnova O.O., Kalitukha I.V., Rodina A.V., Dimitriev G.S., Sapega V.F., Ken O.S., Korenev V.L., Kozyrev N.V., Nekrasov S.V., Kusrayev Y.G. (2023). Optical alignment and optical orientation of excitons in CdSe/CdS colloidal nanoplatelets. Nanomaterials.

[37] Yakovlev D.R., Bayer M., Dyakonov M.I. (2017). Chapter on Coherent spin dynamics of carriers. Spin Physics in Semiconductors.

[38] Hu R.R., Yakovlev D.R., Liang P., Qiang G., Chen C., Jia T., Sun Z., Bayer M., Feng D. (2019). Origin of two larmor frequencies in the coherent spin dynamics of colloidal CdSe quantum dots revealed by controlled charging. J. Phys. Chem. Lett..

[39] Zhang Y.Y., Jiang M.Z., Wu Z., Yang Q., Men Y., Cheng L., Hu R.R., Jia T.Q., Sun Z.R., Feng D.H. (2021). Hyperfine-induced electron-spin dephasing in negatively charged colloidal quantum dots: A survey of size dependence. J. Phys. Chem. Lett..

[40] Feng D.H., Li X., Jia T.Q., Pan X.Q., Sun Z.R., Xu Z.Z. (2012). Long-lived, room-temperature electron spin coherence in colloidal CdS quantum dots. Appl. Phys. Lett..

[41] Feng D.H., Shan L.F., Jia T.Q., Pan X.Q., Tong H.F., Deng L., Sun Z.R., Xu Z.Z. (2013). Optical manipulation of electron spin coherence in colloidal CdS quantum dots. Appl. Phys. Lett..

[42] Tong H.F., Feng D.H., Li X., Deng L., Leng Y.X., Jia T.Q., Sun Z.R. (2013). Room-temperature electron spin generation by femtosecond laser pulses in colloidal CdS quantum dots. Materials.

[43] Li X., Feng D.H., Tong H.F., Jia T.Q., Deng L., Sun Z.R., Xu Z.Z. (2014). Hole surface trapping dynamics directly monitored by electron spin manipulation in CdS nanocrystals. J. Phys. Chem. Lett..

[44] Qiang G., Zhukov E.A., Evers E., Yakovlev D.R., Golovatenko A.A., Rodina A.V., Onushchenko A.A., Bayer M. (2022). Electron spin coherence in CdSe nanocrystals in a glass matrix. ACS Nano.

[45] Stern N.P., Poggio M., Bartl M.H., Hu E.L., Stucky G.D., Awschalom D.D. (2005). Spin dynamics in electrochemically charged CdSe quantum dots. Phys. Rev. B.

[46] Li Y.Q., Steuerman D.W., Berezovsky J., Seferos D.S., Bazan G.C., Awschalom D.D. (2006). Cavity enhanced Faraday rotation of semiconductor quantum dots. Appl. Phys. Lett..

[47] Zhang J., Tang Y., Lee K., Ouyang M. (2010). Tailoring light–matter–spin interactions in colloidal hetero-nanostructures. Nature.

[48] Fumani A.K., Berezovsky J. (2013). Magnetic-field-dependent spin decoherence and dephasing in room-temperature CdSe nanocrystal quantum dots. Phys. Rev. B.

[49] Zhang Z., Jin Z., Ma H., Xu Y., Lin X., Ma G., Sun X. (2014). Room-temperature spin coherence in zinc blende CdSe quantum dots studied by time-resolved Faraday ellipticity. Phys. E Low-Dimens. Syst. Nanostructures.

[50] Feng D.H., Yakovlev D.R., Dubertret B., Bayer M. (2020). Charge separation dynamics in CdSe/CdS core/shell nanoplatelets addressed by coherent electron spin precession. ACS Nano.

[51] Glazov M.M., Sherman E.Y., Dugaev V.K. (2010). Two-dimensional electron gas with spin–orbit coupling disorder. Phys. E Low-Dimens. Syst. Nanostructures.

[52] Bindel J.R., Pezzotta M., Ulrich J., Liebmann M., Sherman E.Y., Morgenstern M. (2016). Probing variations of the Rashba spin–orbit coupling at the nanometre scale. Nat. Phys..

[53] Yugova I.A., Glazov M.M., Ivchenko E.L., Efros A.L. (2009). Pump-probe Faraday rotation and ellipticity in an ensemble of singly charged quantum dots. Phys. Rev. B.

[54] Roth L.M., Lax B., Zwerdling S. (1959). Theory of optical magneto-absorption effects in semiconductors. Phys. Rev..

[55] Semina M.A., Golovatenko A.A., Rodina A.V. (2021). Influence of the spin-orbit split-off valence band on the hole *g*-factor in semiconductor nanocrystals. Phys. Rev. B.

[56] Rodina A.V., Ivchenko E.L. (2020). Theory of single and double electron spin-flip Raman scattering in semiconductor nanoplatelets. Phys. Rev. V.

[57] Karimov O.Z., Wolverson D., Davies J.J., Stepanov S.I., Ruf T., Ivanov S.V., Sorokin S.V., O’Donnell C.B., Prior K.A. (2000). Electron *g*-factor for cubic *Zn*_1-x_*Cd*_x_*Se* determined by spin-flip Raman scattering. Phys. Rev. B.

[58] Oestreich M., Rühle W.W. (1995). Temperature dependence of the electron Landé *g*-factor in GaAs. Phys. Rev. Lett..

[59] Oestreich M., Hallstein S., Heberle A.P., Eberl K., Bauser E., Rühle W.W. (1996). Temperature and density dependence of the electron Landé *g* factor in semiconductors. Phys. Rev. B.

[60] Zawadzki W., Pfeffer P., Bratschitsch R., Chen Z., Cundiff S.T., Murdin B.N., Pidgeon C.R. (2008). Temperature dependence of the electron spin *g* factor in GaAs. Phys. Rev. B.

[61] Hübner J., Döhrmann S., Hägele D., Oestreich M. (2009). Temperature-dependent electron Landé *g* factor and the interband matrix element of GaAs. Phys. Rev. B.

[62] Cragg G.E., Efros A.L. (2010). Suppression of Auger processes in confined structures. Nano Lett..

[63] Glazov M.M. (2012). Coherent spin dynamics of electrons and excitons in nanostructures (a review). Phys. Sol. State.

[64] Smirnov D.S., Glazov M.M. (2012). Spin coherence generation and detection in spherical nanocrystals. J. Phys. Condens. Matter.

[65] Gupta J.A., Awschalom D.D., Efros A.L., Rodina A.V. (2002). Spin dynamics in semiconductor nanocrystals. Phys. Rev. B.

[66] Kirstein E., Kopteva N.E., Yakovlev D.R., Zhukov E.A., Kolobkova E.V., Kuznetsova M.S., Belykh V.V., Yugova I.A., Glazov M.M., Bayer M. (2023). Mode locking of hole spin coherences in CsPb(Cl,Br)_3_ perovskite nanocrystals. Nat. Commun..

[67] Rosen N., Zener C. (1932). Double Stern-Gerlach experiment and related collision phenomena. Phys. Rev..

[68] Chen P., Whaley K.B. (2003). Magneto-optical response of CdSe nanostructures. Phys. Rev. B.

