# Supplementary material for: Coherent Spin Dynamics of Electrons in CdSe Colloidal Nanoplatelets
Source: Nanomaterials (Basel). 2023 Dec 4;13(23):3077. doi: 10.3390/nano13233077 (PMC10708201; doi:10.3390/nano13233077)
Supplement: Supplementary file 1 [file nanomaterials-13-03077-s001.zip › nanomaterials-2665835-supplementary.pdf]

SUPPLEMENTARY MATERIALS

Coherent spin dynamics of electrons in CdSe colloidal nanoplatelets

Sergey R. Meliakov, Vasilii V. Belykh, Ina V. Kalitukha, Aleksandr A. Golovatenko, Alessio Di Giacomo, Iwan Moreels, Anna V. Rodina, Dmitri R. Yakovlev

S1. Dynamics of photoluminescence and differential transmission at T = 5 K and 295 K

The comparison between photoluminescence (PL) dynamics (spectrally integrated over the PL line) and differential transmission dynamics at $T = 5$ K and 295 K is shown in Figure S1(a-f). Both PL and differential transmission dynamics are multi-exponential. The fast component of the transmission and PL exhibits the same decay times of $10-20$ ps. However, the slow transmission component ($> 1-2$ ns) is considerably longer than that of PL ($100-500$ ns). Furthermore, the long dynamics of differential transmission are composed of several components (with decay times of $1-2$ ns and longer than 10 ns) and the amplitude of the longest component becomes negative for decreased detection energy (Figure S1b). On the other hand, at room temperature, such a dependence of differential transmission on spectral energy is not observed, and there is a long decay time of several nanoseconds for all dynamics (Figure S1e).

The difference in the behavior of $\Delta T/T$ and PL dynamics is related to the fact that the PL results from the radiative recombination of excitons, while $\Delta T/T$ is sensitive to the presence of the excitons and charge carriers. $\Delta T/T$ has contributions from bleaching for a large number of carriers, which reduces the optical transition oscillation strength. Another contribution to $\Delta T/T$ comes from the redistribution of oscillator strengths between neutral exciton and charged exciton (trion) transitions, when the photogeneration of charge carriers, which are not bound to excitons, results in the appearance of the trion resonance in the absorption spectrum. This leads to an increase of $\Delta T/T$ at exciton energy and a decrease at trion energy. This redistribution of oscillator strengths explains the change in the sign of $\Delta T/T$ when changing the photon energy (Figure S1b). Thus, the fast component of $\Delta T/T$, similar to PL, results from the recombination of photoexcited carriers. The slow components, including those that appear as a negative background in Figure S1b, correspond to the decay of the concentration of spatially separated charge carriers. In NPLs, a charge separation effect was observed previously, with predominant hole capture to the NPL surface [S1]. positive component with a few ns decay time presumably corresponds to the non-radiative Auger recombination of electrons with holes captured to the surface [S2]. Some holes are separated even further, leaving uncompensated electrons, which presumably give rise to the long-lasting negative component in Figure S1b. At high temperatures, as shown in Figure S1e, the same scenario applies; however, trion and exciton resonances are blurred, and a negative signal is no longer observed at lower photon energies. This indicates that although spatial separation of carriers still occurs, the rate of their recombination is significantly increased. Thus, the observed ns-long spin dynamics obviously correspond to the resident electrons rather than to electrons bound in excitons.

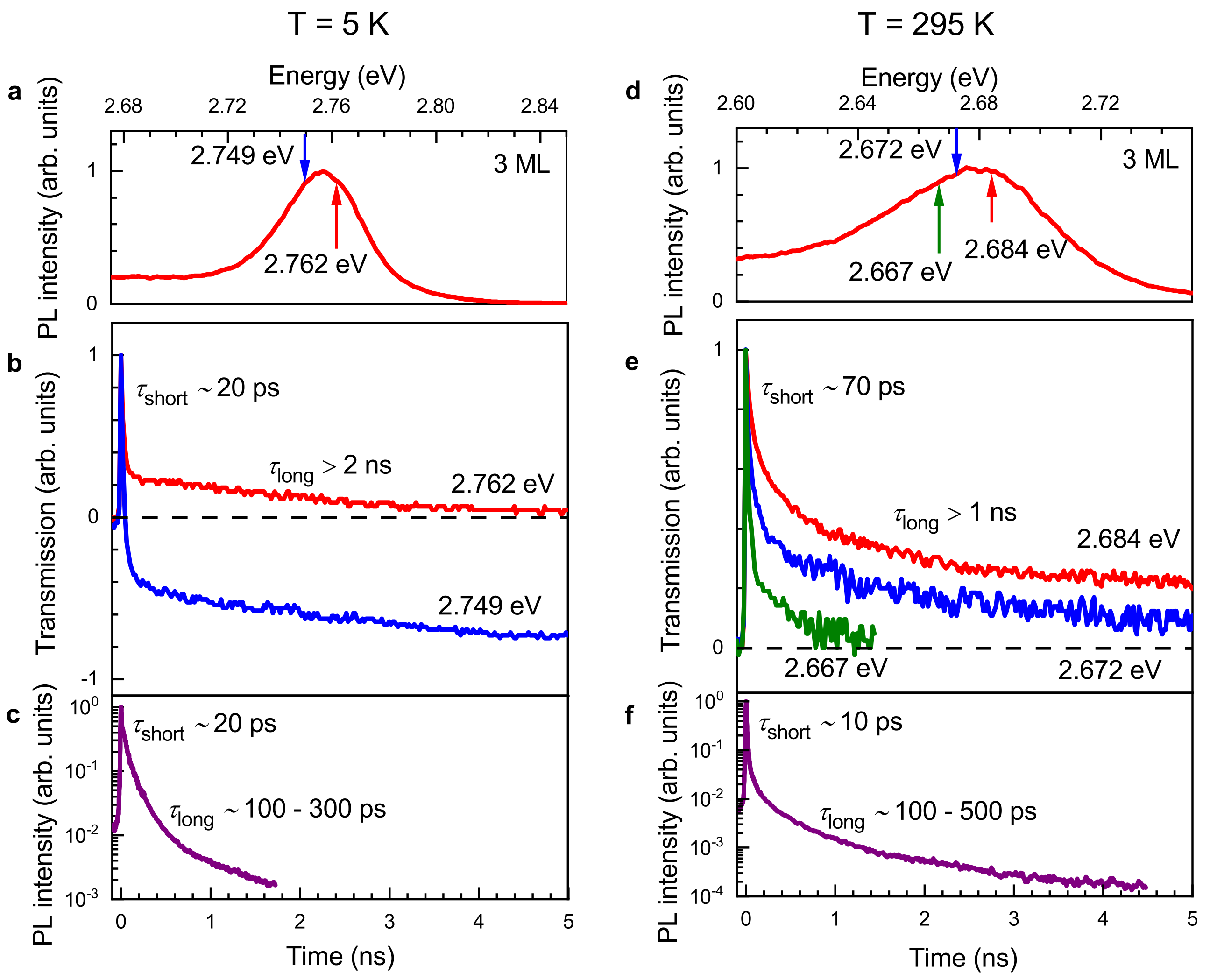

Figure S1. Dynamics of PL and differential transmission in 3 ML CdSe NPLs measured at $T = 5$ K (a-c) and 295 K (d-f). Respective PL spectra are shown in panels (a,d). Here, arrows mark spectral energies, where the differential transmission is measured. PL dynamics in panels (c,f) are spectrally integrated. Note the logarithmic scale for signal intensity in panels (c) and (f) and the linear scale in panels (b) and (e).

S2. Spectral dependences of spin parameters in 3 ML NPLs

Figure S2 shows the spectral dependencies of the spin parameters (g-factor and spin-dephasing time T_2^*) measured by TRFR in 3 ML NPLs. Experiments are performed at $T = 5$ K in a magnetic field of $B = 270$ mT applied in Voigt geometry. Panel (a) shows the PL spectrum and dependence of the FR amplitude on the laser photon energy. At an energy of 2.74 eV, the phase of the oscillations changes by π, which corresponds to the negative amplitudes. The spectral dependence of T_2^* shows the threshold-like behavior changing from 0.6 ns at low energies to 0.3 ns at high energies. The change takes place in the range coinciding with the spin precession phase change. The electron g-factor also shows weak changes around this energy increasing from 1.81 to 1.87, with an increase in the spectral energy. We attribute the changes in the g-factor and T_2^* to the localization of the resident electrons: at low energies, we probe localized electrons, while at higher energies, we probe almost-free electrons.

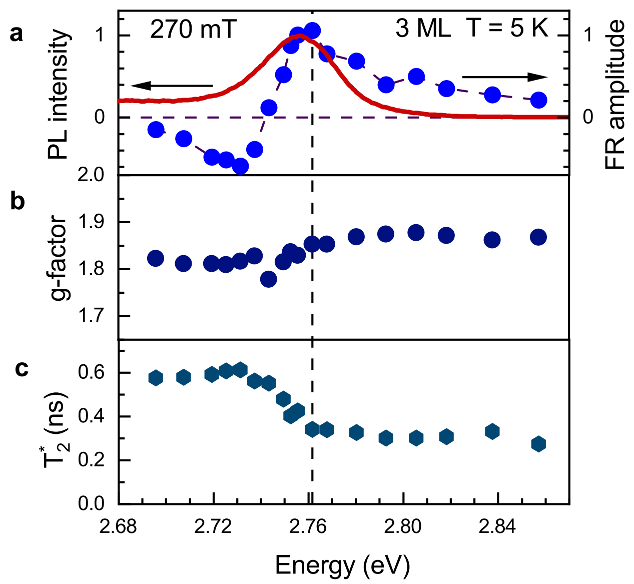

Figure S2. Spectral dependencies of the spin dynamic parameters in 3 ML CdSe NPLs at $T = 5$ K and $B = 270$ mT. (a) PL spectrum (red solid line) and the dependence of the amplitude of the TRFR signal oscillations (blue circles) on laser energy. The vertical dashed line marks the maximum FR amplitude. The spectral dependencies of the g-factor (b) and spin-dephasing time T_2^* (c).

S3. The magnetic field dependence of the spin-dephasing time at room temperature

Figure S3a plots TRFR traces of CdSe NPLs in a solution at room temperature and magnetic field strengths from 0 mT to 330 mT. Fitting with Eq. (2) allows us to determine the spin-dephasing time T_2^*, which decreases from approximately 0.5 ns at the minimal magnetic field to 0.2 ns at the maximal field. We can rewrite Eq. (3) as

$$\frac{1}{T_2^*} = \frac{1}{T_2^*(0)} + \frac{\Delta g}{g}\omega_L \,. \tag{S1}$$

The dependence of time T_2^* on the Larmor precession frequency (which serves as a measure of the magnetic field) is shown in Figure S3b. Fitting the experimental points using Eq. (S1) yields $\Delta g = 0.08$ for 3 ML NPLs, which agrees well with the low-temperature result, $\Delta g = 0.06$. For 4 ML NPLs, $\Delta g = 0.17$ is twice larger than that for 3 ML NPLs.

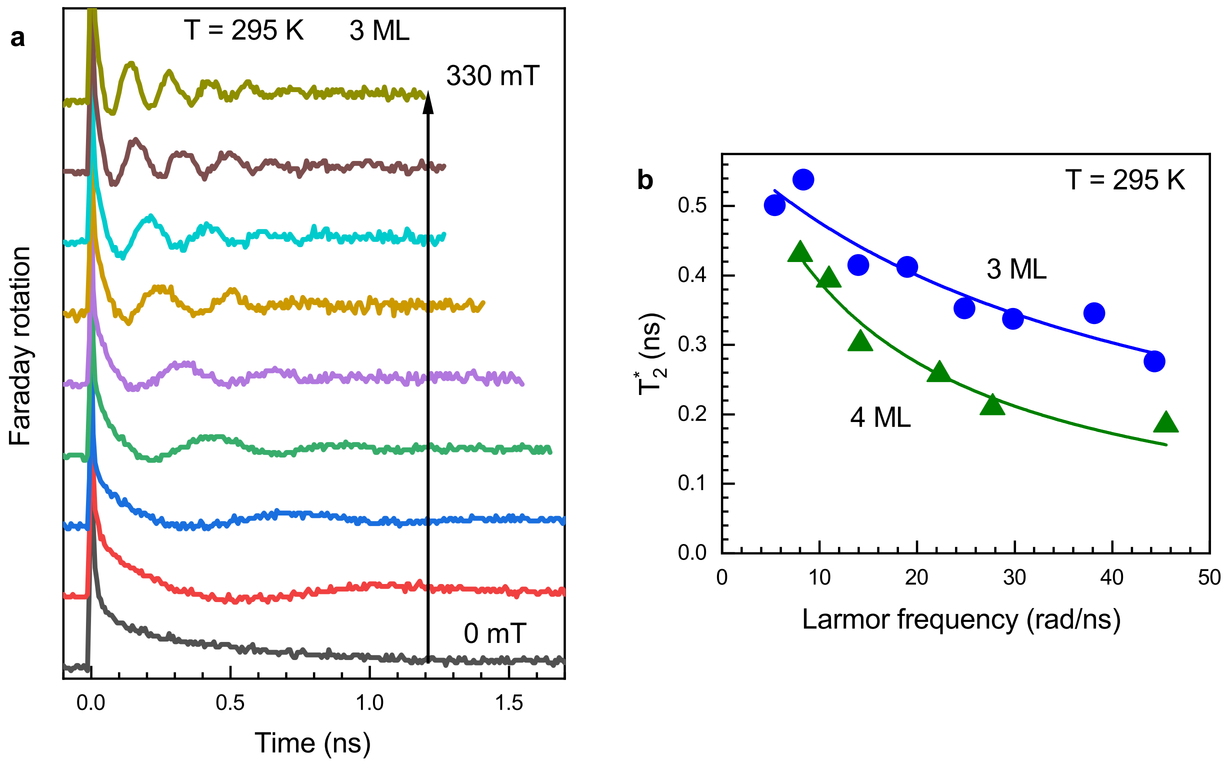

Figure S3. (a) Spin dynamics measured in 3 ML NPLs at room temperature in different magnetic fields. Laser photon energy is 2.638 eV (470 nm). (b) Dependence of the spin-dephasing time T_2^* for 3 ML (blue circles) and 4 ML (green triangles) NPLs on the Larmor frequency. The solid lines show fits of the data using Eq. (2) with parameters $\Delta g = 0.08$ and $\Delta g = 0.17$, respectively.

S4. Longitudinal spin relaxation times T_1

Table S1 shows the longitudinal spin relaxation time T_1 and the inhomogeneous spin-dephasing time T_2^* at $T = 10$ K and $T = 295$ K obtained from fitting the TRFR traces using Eq. (4) in the main text. The magnetic field of 50 mT is directed at an angle $\alpha = 35°$ to the substrate normal. Time T_2^* decreases with temperature from 1 ns to 0.7 ns for 3 ML NPLs and from 1.4 ns to 0.3 ns for 4 ML NPLs. Longitudinal relaxation has two components: T_1^{short} and T_1^{long}. Time T_1^{short} decreases with temperature from 1.9 ns to 0.7 ns for 3 ML NPLs and from 6.7 ns to 1.1 ns for 4 ML NPLs. T_1^{long} is always longer than 10 ns for NPLs of both thicknesses, even at room temperature.

	$T = 10$ K			$T = 295$ K		
	T_2^* (ns)	T_1^{short} (ns)	T_1^{long} (ns)	T_2^* (ns)	T_1^{short} (ns)	T_1^{long} (ns)
3 ML	1	1.9	$\gg 10$	0.7	0.7	$\gtrsim 10$
4 ML	1.4	6.7	$\gg 10$	0.3	1.1	$\gtrsim 10$

Table S1. Longitudinal spin relaxation times T_1 and inhomogeneous ensemble spin-dephasing times T_2^* for the 3 ML and 4 ML CdSe NPLs measured at $T = 10$ K and 295 K. The magnetic field of 50 mT tilted by $\alpha = 35°$ to the substrate normal.

S5. Trion binding energies

Splitting between the exciton (X) and negative trion (T) lines, as shown in Figure 1a in the main text, corresponds to the trion binding energy. These splittings are: 20 ± 1 meV for 5 ML, 24 ± 1 meV for 4 ML, and 42 ± 3 meV for 3 ML CdSe NPLs. Their comparison with trion binding energies from Ref. [S3] is shown in Figure S4.

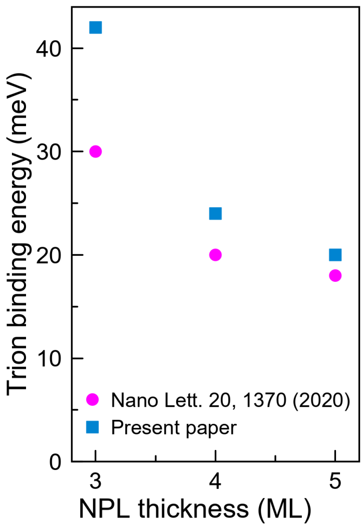

Figure S4. Splitting between the exciton (X) and negative trion (T) lines as a function of NPL thickness (blue squares). Data for the trion binding energies from Ref. [S3] are shown by magenta circles.

S6. Spin–flip Raman scattering in 4 ML NPLs

Figure S5 shows the SFRS spectra of 4 ML NPLs. Figure S5a demonstrates co- and cross-circular polarized SFRS, while Figure S5b demonstrates co- and cross-linear polarized SFRS; both are measured under excitation at $E_{\mathrm{exc}} = 2.541$ eV in the Faraday magnetic field $B = 5$ T. Stokes (positive Raman shifts) and anti-Stokes (negative Raman shifts) areas of the spectra contain lines with energy around ± 0.45 meV. These lines are attributed to the electron spin–flip Raman scattering. The electron spin–flip line is more pronounced in co-circular polarizations (σ^+ excitation and σ^+ detection) and in cross-linear polarization configurations (horizontal polarization of excitation and vertical polarization of detection).

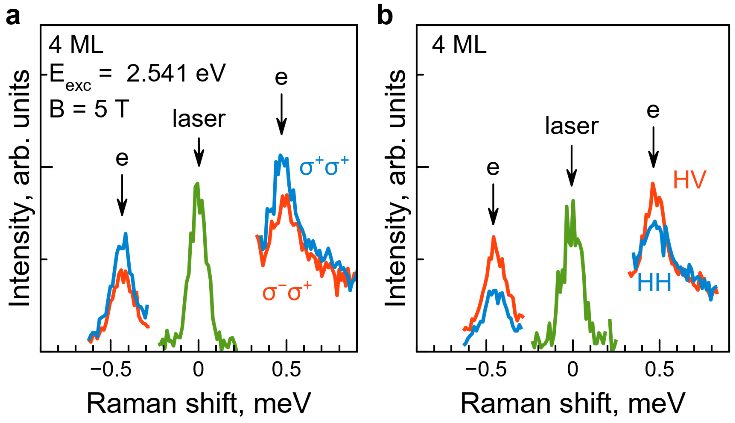

Figure S5. SFRS in 4 ML NPLs measured in (a) circular and (b) linear polarizations. The magnetic field $B = 5$ T is applied in Faraday geometry. $T = 2$ K. Polarization properties of SFRS agree with those of the 3 ML sample (Figure 4a in the main text) and with the selection rules described in Ref. [S11].

S7. Calculation of the electron g-factor dispersion caused by the dispersion of NPL lateral sizes

To identify the origin of the g-factor spread evaluated from the magnetic field dependence of the spin-dephasing time T_2^* (Figure 2 in the main text), we calculated the electron g-factor in NPLs of fixed thicknesses but different widths, $5, 7, 9$ nm. The length of the NPL is about 30 nm and its dispersion does not play any role in the renormalization of the g-factor. For this purpose, we use the kp-model described in Ref. [S4]. The result of the calculation and the comparison with the experimental data are shown in Figure 5. The dashed lines in Figure 5 show the results of the calculation for infinite quantum box confinement potential, and the solid lines denote the harmonic oscillator potential. These two potentials differ by the localization of the electron wave function near the center of the NPL. For the infinite quantum box potential calculated g-factor, one can see that values are far below the experimental data points. We note that spherical CdSe NC calculations with this confinement potential are in excellent agreement with the experimental g-factor values [S5]. For the infinite quantum box confinement potential, we find $\Delta g = 0.015$ for 3 ML NPLs and $\Delta g = 0.02$ for 4 ML NPLs. These values are several times smaller as compared to the Δg values determined experimentally.

For the harmonic oscillator potential (solid lines), we find better agreement between the calculated and experimental g-factors. The harmonic oscillator potential provides stronger localization near the center of the NPL. We suggest that it could be related to the dielectric confinement effect, resulting in the repulsion of the electron from its image charge. However, due to the larger quantization energy of the electron, the effect of the NPL width dispersion on the spread of electron g-factors becomes even smaller, as one can see in Figure 5. From that, we conclude that the dispersion of NPL lateral sizes is not sufficient to explain the origin of T_2^* observed experimentally. Likely, the spread of g-factors is caused by the variation of dielectric confinement or strain effects for the NPLs in the ensemble. Moreover, the calculated g-values for the parabolic potential confining the electron better describe the experimental data than those for the box-like potential profile. This may point to the specific localization of resident electrons in the NPL, and the spread of g-factors observed in the TRFR can be attributed to fluctuations in the localization conditions.

The calculation results shown in Figure 5 neglect the quantization of the electron along the length of an NPL. In Figure S6, we show the difference between the dependencies of the g-factor on the NPL thickness for NPLs with a width of 9 nm and either infinite or 30 nm length. The g-factor difference is determined as $g(l = \infty, w = 9\text{ nm}) - g(l = 30\text{ nm}, w = 9\text{ nm})$. The solid line corresponds to the harmonic oscillator potential; the dashed line corresponds to the infinite quantum box potential. One can see that the g-factor difference is negligible, and the 30 nm NPL length can be safely treated as infinite. The influence of electron quantization along the NPL width is shown by blue lines, which are calculated as $g(l = \infty, w = \infty) - g(l = \infty, w = 9\text{ nm})$. One can see that, even in this case, the g-factor difference is smaller than the experimental Δg values.

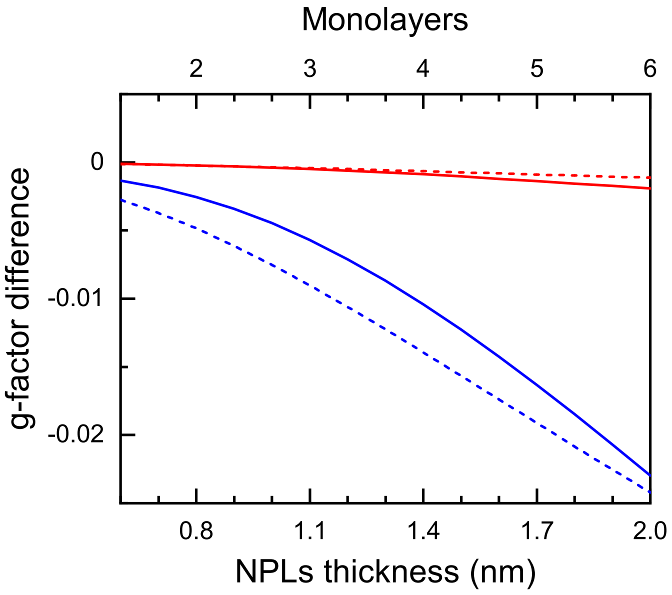

Figure S6. The difference in the electron g-factor for NPLs of different thicknesses and a 9 nm width, having either infinite or 30 nm length, is depicted by the red lines. The difference between NPLs of 9 nm width and infinite length and NPLs of infinite width and length is illustrated by the blue lines. Solid lines correspond to the harmonic oscillator localization potential, while the dashed line corresponds to the infinite quantum box localization potential.

S8. Theory of TRFR in CdSe NPLs

A detailed microscopic analysis of the TRFR for an ensemble of singly charged (In,Ga)As epitaxial quantum dots can be found in Refs. [S6, S7]. The microscopic mechanism of the TRFR proposed in Ref. [S6] is based on the excitation of a negatively charged trion (a heavy hole plus two electrons with opposite spins) in a quantum dot charged by one resident electron. The idea of the trion mechanism is as follows: A circularly polarized pump pulse directed along the quantization axis of a negatively charged quantum dot excites the singlet state of trions in quantum dots where the electron has a spin opposite to that of the photocreated electron. This occurs due to strict selection rules for the interaction of circularly polarized photons with heavy hole-based trions. After the fast spin relaxation of a heavy hole in a trion, followed by the radiative recombination of the trion, the electron spin polarization in these quantum dots becomes random. However, the entire ensemble becomes spin-polarized after excitation due to the quantum dots having electron spins parallel to those of the photocreated electrons, which remain unaffected by the excitation pulse due to the aforementioned selection rules.

Further, the initialized electron spin polarization precesses in the transverse magnetic field. When there is electron spin polarization along the quantum dot's quantization axis (z-axis), the probe pulse linearly polarized along the x-axis of the laboratory frame creates dielectric polarization along the y-axis of the laboratory frame in the quantum dot layer. This dielectric polarization along the y-axis results in the appearance of the y-polarized electric field component for the transmitted probe pulse. In other words, the rotation of the polarization plane of the transmitted probe pulse occurs.

Later, the trion mechanism was extended for the case of spherical colloidal nanocrystals with a zinc blende crystal structure [S8]. Due to zero splitting between the heavy and light hole states in these NCs, a circularly polarized photon excites a coherent superposition of trions formed by the heavy hole and light hole. The most striking difference from the epitaxial quantum dots considered in Ref. [S6] involves theoretically predicted sign-reversal Rabi oscillations of the electron spin polarization created by the pump pulse [S8].

Colloidal CdSe nanoplatelets are analogous to epitaxial quantum dots due to close lateral dimensions and the presence of a quantization axis, which leads to a large $200-300$ meV [S9] splitting between heavy holes and light holes. However, the quantization axis in the NPL ensemble is oriented randomly or may have several preferential orientations [S10, S11]; in contrast, epitaxial quantum dots have strict orientation. The random orientation is also valid for spherical colloidal wurtzite CdSe nanocrystals with a large (compared to the Zeeman splitting) splitting between heavy and light holes induced by the crystal field (≈ 25 meV). These nanocrystals have been extensively studied by TRFR since 2002 [S12, S13, S5].

In such colloidal ensembles of NPLs or NCs, the direction of the magnetic field and the direction of the incident light do not have strictly defined orientations with respect to the anisotropy axis. This does not allow one to provide a direct analysis of the experimental data for colloidal CdSe NPLs or CdSe NCs using the theory developed in Ref. [S6]. Partially, the random orientation effect of NCs is considered in Ref. [S14] for lead halide perovskite NCs. However, this paper does not consider the selection rules for optical transitions involving a heavy hole, which are of decisive importance in the case of CdSe NPLs.

Here, we extend the theory from Ref. [S6] and analyze the difference in pump–probe Faraday rotation studies for: (i) an ensemble of NPLs with two predominant orientations (lying flat and standing on the edge, see Figure S7a); (ii) an ensemble of NPLs forming a "fish scale" stacking (see Figure S7b). It is assumed that the deviation of the NPL plane in the ensemble from the plane of the substrate is uniformly distributed in the range from 0 to 30 degrees. (iii) An ensemble of NPLs with random orientations of the anisotropy axis; see Figure S7c. The essential approximations of our theoretical model are as follows. We assume that the observed TRFR signal is based on the trion mechanism. This assumption is consistent with the experimental fact that the maximum of the TRFR signal coincides with the maximum of the trion PL band. Next, we consider singlet negative trions formed by two electrons and a heavy hole. For these trions, the interaction of carriers with light is determined by a two-dimensional electric dipole lying in the NPL plane. The trion dipole moment directed out of the NPL plane is equal to zero, i.e. there is no interaction of trions with the corresponding light component, as assumed in Ref. [S6].

Coordinate systems

We start by finding the correspondence between the coordinate system associated with the experimental setup (laboratory frame) and the coordinate system associated with the nanoplatelet (NPL frame). We introduce the laboratory frame axes $X = (x, y, z)$ and the NPL frame axes $X' = (x', y', z')$. The direction of the pump and probe laser pulses coincides with the z-axis of the laboratory frame. The laboratory frame and the NPL frame are related by $X' = RX$, with the matrix R:

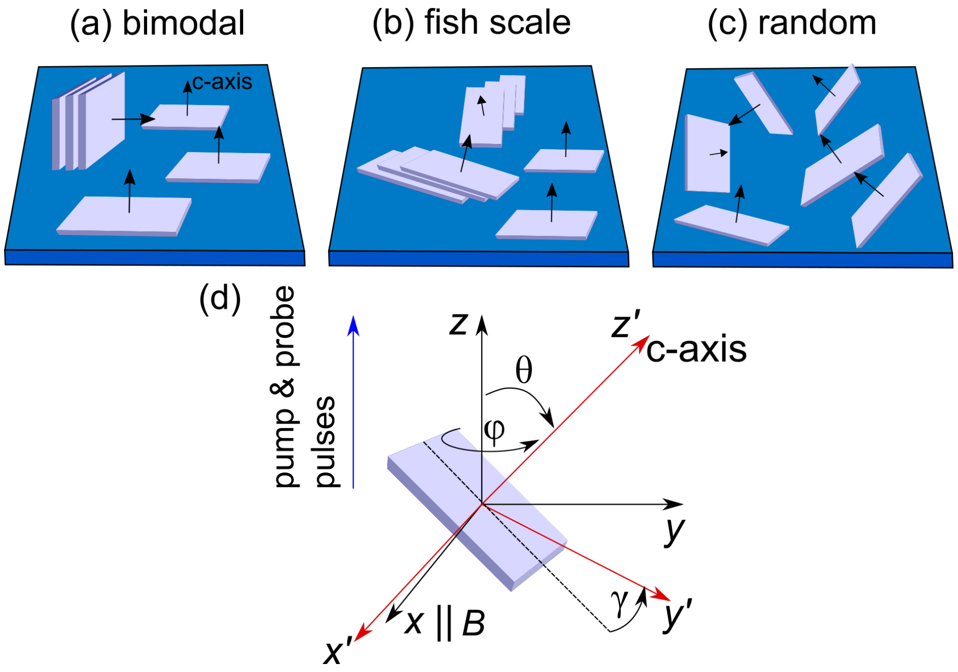

Figure S7. Schematics of nanoplatelet ensembles with different types of orientation on the substrate: (a) bimodal distribution with two orientations, (b) "fish scale" stacking, and (c) random orientation. Panel (d) shows the relative orientation of the laboratory and NPL frames. Pump and probe pulses are directed along the z-axis. The magnetic field is directed along the x-axis. Angles φ, θ, γ are nutation, precession, and intrinsic angles for the $z-y-z$ Euler rotation sequence.

$$R = \begin{pmatrix} \cos\varphi\cos\gamma\cos\theta - \sin\varphi\sin\gamma & \cos\gamma\cos\theta\sin\varphi + \cos\varphi\sin\gamma & -\cos\gamma\sin\theta \\ -\cos\gamma\sin\varphi - \cos\varphi\cos\theta\sin\gamma & \cos\varphi\cos\gamma - \cos\theta\sin\varphi\sin\gamma & \sin\theta\sin\gamma \\ \cos\varphi\sin\theta & \sin\varphi\sin\theta & \cos\theta \end{pmatrix}$$

Matrix R corresponds to a $z-y-z$ rotation sequence transforming the laboratory frame into the NPL frame. The Euler precession (θ) and nutation (φ) angles are the polar and azimuthal angles of the NPL anisotropy axis z' in the laboratory frame, so that the first two rotations completely define the random orientation of the NPL c-axis. The third rotation around the c-axis by the intrinsic angle γ is added for the convenience of calculations. It is chosen to satisfy the condition that the y' axis of the NPL frame is lying in the yz plane of the laboratory frame. One can choose the y'-axis in such a way if the NPLs are axially symmetric with respect to the quantization axis, or if the in-plane anisotropy of the NPLs does not strongly affect the energy states of electrons and trions. Under this assumption, one can express the angle γ in terms of the angles θ and φ using the condition that the projection of the x-axis onto the y'-axis is equal to zero, i.e., $\cos\gamma\sin\varphi = -\cos\varphi\cos\theta\sin\gamma$. We note that the final results do not depend on the angle γ, which confirms the validity of our approach.

For the chosen coordinate systems, the magnetic field directed along the x-axis of the laboratory frame (Voigt geometry) $B_x = B$ has the following components in the NPL frame:

$$\begin{aligned} B_{x'} &= B(\cos\varphi\cos\gamma\cos\theta - \sin\varphi\sin\gamma) = B\sqrt{1-\cos^2\varphi\sin^2\theta}, \\ B_{y'} &= 0, \\ B_{z'} &= B\cos\varphi\sin\theta. \end{aligned} \tag{S2}$$

We also need to find projections on the NPL frame for the circularly polarized pump pulse directed along the z-axis of the laboratory frame. We further consider only the σ^+ polarized pump pulse. The approach for the σ^- polarized pump pulse can be made in a similar way [S6]. The σ^+ polarized pump pulse in the laboratory frame is described by $\boldsymbol{E}_{\sigma^+}(\boldsymbol{r},t) = E(\boldsymbol{r},t)(\boldsymbol{o}_x + i\boldsymbol{o}_y)/\sqrt{2}$, where $\boldsymbol{o}_{x,y}$ are unit vectors directed along the x and y-axes of the laboratory frame. The amplitude $E(\boldsymbol{r},t)$ is proportional to $e^{i\omega_p t}$ with ω_p being the frequency of photons in the pump pulse. In

the NPL frame, the pump pulses have both $\sigma^{\pm}$ components with the following amplitudes:

$$\boldsymbol{E}_{\sigma^+}(\boldsymbol{r},t) = E(\boldsymbol{r},t)\frac{1+\cos\theta}{2}e^{i(\varphi+\gamma)}\frac{\boldsymbol{o}_{x'}+i\boldsymbol{o}_{y'}}{\sqrt{2}}, \tag{S3}$$

$$\boldsymbol{E}_{\sigma^-}(\boldsymbol{r},t) = -E(\boldsymbol{r},t)\frac{1-\cos\theta}{2}e^{i(\varphi-\gamma)}\frac{\boldsymbol{o}_{x'}-i\boldsymbol{o}_{y'}}{\sqrt{2}}. \tag{S4}$$

Here, we neglected the linearly polarized component of the pump pulse $\boldsymbol{E}_z(\boldsymbol{r},t)$ in the NPL frame since the trion dipole moment along the z'-axis is equal to zero.

Finally, we find the components $E_{x',y',z'}$ of the probe pulse $\boldsymbol{E}_{\mathrm{pr}}(\boldsymbol{r},t) = E(\boldsymbol{r},t)\boldsymbol{o}_x$ linearly polarized along the x-axis of the laboratory frame. Components $E_{x',y',z'}$ can be written in a similar way to the magnetic field components:

$$\begin{aligned}
&\boldsymbol{E}_{x'}(\boldsymbol{r},t) = E(\boldsymbol{r},t)\sqrt{1-\cos^2\varphi\sin^2\theta}\,\boldsymbol{o}_{x'}, \\
&\boldsymbol{E}_{y'}(\boldsymbol{r},t) = 0, \\
&\boldsymbol{E}_{z'}(\boldsymbol{r},t) = E(\boldsymbol{r},t)\cos\varphi\sin\theta\,\boldsymbol{o}_{z'}.
\end{aligned} \tag{S5}$$

Again, the zero dipole moment of the trion transition allows us to consider only the $E_{x'}$ component of the electric field. Hence, for the probe pulse, the only important effect arising from the arbitrary orientation of the NPL quantization axis is the renormalization of its $E_{x'}$.

Initialization of electron spin polarization

Following Ref. [S6], we consider the action of a short σ^+ polarized laser pulse with frequency ω_p close to the trion resonance frequency, ω_0. The pulse duration τ_p is considered short compared to the spin relaxation times of the electron and trion, the trion radiative lifetime, and the spin precession period of an electron and a hole in an external magnetic field. The interaction of an NPL with the electromagnetic wave is described by the Hamiltonian [S6]:

$$\hat{V}(t) = -\int [\hat{d}_+(\boldsymbol{r})E_{\sigma_+}(\boldsymbol{r},t) + \hat{d}_-(\boldsymbol{r})E_{\sigma_-}(\boldsymbol{r},t)]d^3r, \tag{S6}$$

where $\hat{d}_\pm(\boldsymbol{r}) = [\hat{d}_x(\boldsymbol{r}) \pm i\hat{d}_y(\boldsymbol{r})]/\sqrt{2}$ denotes the circularly polarized components of the dipole moment density operator, and $E_{\sigma_\pm}(\boldsymbol{r},t)$ denotes the circularly polarized components of the electric field of a quasi-monochromatic electromagnetic wave.

The pump pulse induces the optical transition between the electron state and the trion state. As a result, the coherent superposition of these states is created. It is convenient to introduce a four-component wave function:

$$\Psi = (\psi_{1/2}, \psi_{-1/2}, \psi_{3/2}, \psi_{-3/2}), \tag{S7}$$

where the $\pm 1/2$ subscripts denote the electron spin projection and $\pm 3/2$ refers to the spin projection of a hole in a negative trion. The electron spin polarization is expressed in terms of $\psi_{\pm 1/2}$ as follows:

$$S_{z'} = (|\psi_{1/2}|^2 - |\psi_{-1/2}|^2)/2, \quad S_{x'} = \mathrm{Re}(\psi_{1/2}\psi^*_{-1/2}), \quad S_{y'} = -\mathrm{Im}(\psi_{1/2}\psi^*_{-1/2}). \tag{S8}$$

In the rotating-wave approximation, the action of a short σ^+ pump pulse on the negatively charged NPL is described by the following equations:

$$\begin{aligned}
&i\hbar\dot{\psi}_{3/2} = \hbar\omega_0\psi_{3/2} + V_+(t)\psi_{1/2}, \\
&i\hbar\dot{\psi}_{1/2} = V_+^*(t)\psi_{3/2}, \\
&i\hbar\dot{\psi}_{-3/2} = \hbar\omega_0\psi_{-3/2} + V_-(t)\psi_{-1/2}, \\
&i\hbar\dot{\psi}_{-1/2} = V_-^*(t) + V_+^*\psi_{-3/2}.
\end{aligned} \tag{S9}$$

Here, $\dot{\psi} = \partial\psi/\partial t$, $V_\pm = -\int d(\boldsymbol{r})E_{\sigma^\pm}(\boldsymbol{r},t)e^{i(\varphi\pm\gamma)}(\cos\theta \pm 1)/2$, ω_0 is the trion resonance frequency. From Eq. (S9), one can see that the σ^+ pump pulse creates both trion states $\psi_{\pm 3/2}$, in contrast to the case of epitaxial QDs [S6]. The effective transition dipole $d(\boldsymbol{r})$ equals [S6]:

$$d(\boldsymbol{r}) = \langle 1/2|\,\hat{d}_-(\boldsymbol{r})\,|3/2\rangle = \langle -1/2|\,\hat{d}_+(\boldsymbol{r})\,|-3/2\rangle = -i\frac{ep_{cv}}{\omega_0 m_0}\mathbf{F}(\boldsymbol{r},\boldsymbol{r}), \tag{S10}$$

which is the matrix element of the operators $\hat{d}_{\pm}(\boldsymbol{r})$ calculated between the wave functions of the valence band $|\pm 3/2\rangle$ and the conduction band, $|\pm 1/2\rangle$, all taken in the electron representation. In Eq. (S10), e is the electron charge, m_0 is the free electron mass, and $p_{cv} = \langle \mathbf{S}| \hat{p}_x |\mathbf{X}\rangle = \langle \mathbf{S}| \hat{p}_y |\mathbf{Y}\rangle = \langle \mathbf{S}| \hat{p}_z |\mathbf{Z}\rangle$ is the interband matrix element of the momentum operator taken between the conduction- and valence-band Bloch functions at the Γ point of the Brillouin zone, $\mathbf{S}$, and $(\mathbf{X}, \mathbf{Y}, \mathbf{Z})$, respectively.

However, the pump pulse does not mix all states of the electron–trion system, only pairs $\psi_{1/2}/\psi_{3/2}$ and $\psi_{-1/2}/\psi_{-3/2}$. Thus, one can find the relationship between the electron components of the wave function Ψ after $(\psi_{\pm 1/2}(\infty))$ and before $(\psi_{\pm 1/2}(-\infty))$ the pump pulse action in the way proposed in Ref. [S6]. The solution has the following form:

$$\psi_{\pm 1/2}(\infty) = Q_{\pm} e^{i\Phi_{\pm}} \psi_{\pm 1/2}(-\infty). \tag{S11}$$

Here, the real coefficients $Q_{\pm}$ satisfy the condition $0 \le Q_{\pm} \le 1$, and phases $\Phi_{\pm}$ can be chosen in the range between $-\pi$ and π. The explicit forms of $Q_{\pm}$ and $\Phi_{\pm}$ depend on the parameters of the pump pulse. In what follows, we consider smooth pulses of the shape proposed by Rosen and Zener [S15]:

$$f(t) = \frac{\mu}{\cosh(\pi t/\tau_p)}, \tag{S12}$$

where the coefficient μ is a measure of the pulse electric field strength, and τ_p is the pulse duration. The effective area of the pulse is $\Theta = 2\mu\tau_p$. For the Rosen–Zener pump pulse parameters, $Q_{\pm}$ and $\Phi_{\pm}$ take a form similar to that in Ref. [S8]:

$$Q_{\pm} = \sqrt{1 - \frac{\sin^2(\Theta_{\pm}/2)}{\cosh^2 \pi\chi}}, \tag{S13}$$

$$\Phi_{\pm} = \arg\left[\frac{\Gamma^2(\frac{1}{2} - i\chi)}{\Gamma(\frac{1}{2} - \frac{\Theta_{\pm}}{2\pi} - i\chi)\Gamma(\frac{1}{2} + \frac{\Theta_{\pm}}{2\pi} - i\chi)}\right], \tag{S14}$$

where $\Theta_{\pm} = \Theta(1 \pm \cos\theta)/2$, and $\chi = (\omega_p - \omega_0)\tau_p/2\pi$ denotes the dimensionless detuning between the pump pulse and trion frequencies.

The electron spin polarization components after the pump pulse are as follows:

$$\begin{aligned} S^+_{z'} &= \frac{Q_+^2 - Q_-^2}{4} + \frac{Q_+^2 + Q_-^2}{2} S^-_{z'}, \\ S^+_{x'} &= Q_+ Q_- (S^-_{x'} \cos(\Phi_+ - \Phi_-) + S^-_{y'} \sin(\Phi_+ - \Phi_-)), \\ S^+_{y'} &= Q_+ Q_- (S^-_{y'} \cos(\Phi_+ - \Phi_-) - S^-_{x'} \cos(\Phi_+ - \Phi_-)). \end{aligned} \tag{S15}$$

These equations are similar to the equations for spherical NCs from Ref. [S8], and for $\theta = 0$, which is the case of epitaxial quantum dots, $Q_- = 1$ and $\Phi_- = 0$; thus, we arrive at Eq. 16(a-c) from Ref. [S6].

Since we are interested in the ensemble of NPLs, which differ by their spin polarizations $S^-_{x',y',z'}$ before the arrival of the pump pulse, averaging over all possible spin polarizations $S^-_{x',y',z'}$ should be performed. The averaging results in $\tilde{S}^-_{x',y',z'} = 0$. Thus, after the pump pulse, the spin polarization components for an ensemble of NPLs are:

$$\tilde{S}^+_{z'} = \frac{Q_+^2 - Q_-^2}{4}, \quad \tilde{S}^+_{x'} = 0, \quad \tilde{S}^+_{y'} = 0. \tag{S16}$$

Larmor precession in magnetic field

In our consideration of the spin precession in the magnetic field, we use the conventional assumption [S6, S8] that the hole-in-trion spin loses its orientation fast, compared with the radiative lifetime of the trion. In this case, the electron remaining after the trion recombination is completely depolarized and the electron spin polarization is determined by equation (S16). In the magnetic field, the electron spin polarization oscillates according to the Bloch equation:

$$\dot{\boldsymbol{S}} + \boldsymbol{S} \times \boldsymbol{\Omega} + \frac{\boldsymbol{S}}{\tau_{s,e}} = 0. \tag{S17}$$

where $\boldsymbol{S} = (S_{x'}, S_{y'}, S_{z'})$ and $\boldsymbol{\Omega} = (\Omega_{x'}, \Omega_{y'}, \Omega_{z'})$. $\Omega_{x',y'} = g_\perp \mu_B B_{x',y'}/\hbar$ and $\Omega_{z'} = g_{||}\mu_B B_{z'}/\hbar$, where $g_{\perp,||}$ are electron g-factors in the direction perpendicular and parallel to the NPL quantization axis, respectively.

For the magnetic field directed along the x-axis of the laboratory frame, and under the condition that the initial spin polarizations after the pump pulse are $\tilde{S}^+ = (0, 0, \tilde{S}^+_{z'})$, the spin precession is described by the following equation:

$$\tilde{S}_{z'}(t) = \tilde{S}^+_{z'} \left(\frac{\Omega^2_{z'}}{\Omega^2} + \frac{\Omega^2_{x'}}{\Omega^2} \cos \Omega t \right) \exp(-t/\tau_{s,e}). \tag{S18}$$

This equation is written with the assumption of the isotropic spin relaxation time $\tau_{s,e}$.

Electron spin readout

The time evolution of the electron spin polarization is detected by the linearly polarized probe pulse. As we have shown above, the tilt of the NPL anisotropy axis with respect to the probe pulse direction results in the renormalization of the effective electric field in the NPL frame. The renormalized electric field component $E_{x'}$ induces electric polarization along the x' and y' axes of the NPL, as shown in Ref. [S6]. The polarization $\delta P_{x'}$ along the x' axis is proportional to the population of electron state $n_e = |\psi_{1/2}|^2 + |\psi_{-1/2}|^2$. The polarization $\delta P_{y'}$ along the y' axis is proportional to the electron spin polarizations $\tilde{S}_{z'}(t)$. As the TRFR signal is determined by the polarization induced along the y-axis of the laboratory frame, we project polarizations $\delta P_{x'}$ and $\delta P_{y'}$ on this axis.

Since we only consider the electron contribution to the TRFR signal, assuming fast trion recombination, the value of n_e remains constant. Thus, the $\delta P_{x'}$ in each NPL is also constant. Integration over all possible orientations of the NPL quantization axis shows that the contribution from $\delta P_{x'}$ to the total polarization δP_y from all NPLs equals zero. It is an expected result since the presence of resident electrons with zero net spin polarization should not affect the polarization plane of the probe pulse. In the case of the $\delta P_{y'}$ polarization component, we find that its contribution to the δP_y polarization is proportional to $\tilde{S}_{z'}(t) \cos\theta$. The total polarization δP^{ens}_y from the ensemble of NPLs can be found by integration over the random distribution of NPL orientations:

$$\delta P^{\text{ens}}_y(\boldsymbol{r}, t) = -\frac{S^{\text{ens}}_z}{\hbar} d^*(\boldsymbol{r}) \int d^3r' \int_{-\infty}^{t} dt' e^{i\omega_0(t'-t)} d(\boldsymbol{r}') \times E^{\text{pr}}_x(\boldsymbol{r}', t') + c.c., \tag{S19}$$

$$S^{\text{ens}}_z = \int_{\varphi=0}^{2\pi} \int_{\theta=0}^{\pi/2} \tilde{S}_{z'}(t) \cos\theta \sin\theta d\theta d\varphi. \tag{S20}$$

The obtained result is identical to the one in Ref. [S6], with one exception—that the spin polarization S^{ens}_z is averaged over all possible orientations of NPLs in the ensemble.

We calculated the time evolution of S^{ens}_z for three types of NPL ensembles, as shown in Figure S7. First, we note that results obtained for a bimodal distribution reproduce the results of Ref. [S6] for epitaxial quantum dots. This is because of the zero spin polarization $\tilde{S}_{z'}$ created by the pump pulse in vertically standing NPLs, which do not contribute to TRFR. On the other hand, the sub-ensemble of NPLs lying flat on the substrate is identical to the ensemble of epitaxial QDs. The bimodal ensemble of NPLs exhibits typical Rabi oscillations of the spin polarization created by the pump pulse (see Figure S8, black lines). The TRFR signal for the bimodal ensemble precesses at the transverse frequency $\Omega_x = g_\perp \mu_B B/\hbar$, and its amplitude remains constant for $\tau_{s,e} \to \infty$.

Let us consider now an ensemble of NPLs with "fish scale" stacking. We model this ensemble by taking the uniform dispersion of angle θ within 30 degrees. The dependence of the initial spin polarization on the pump pulse area is similar to the one for flat-lying NPLs, with one exception—that for very large pump areas, there are regions with opposite signs of electron spin polarization (see Figure S8, blue lines). The time evolution of spin polarization for the "fish scale" ensemble shows slow damping of the precession amplitude in the absence of spin relaxation $\tau_{s,e} \to \infty$ (see Figure S9a). The reason is the spread of the precession frequencies Ω in the ensemble with $g_\perp \neq g_{||}$. The precession frequency is slightly smaller compared to transverse frequency Ω_x (compare $g_{\text{eff}} = 1.87$ and $g^{\text{SFRS}}_\perp = 1.88$). The extracted value of g_{eff} does not depend on the magnetic field within the studied range.

Finally, we consider an ensemble of randomly oriented NPLs. The most striking feature of this ensemble is the sign-reversal dependence of the initialized spin polarization on the pump pulse area (see Figure S8, red lines). As with the case of the "fish scale" ensemble, we find a decrease in the precession amplitude with time, which is more pronounced in the randomly oriented ensemble (compare Figures S9a and S9b). The typical spin-dephasing time due to this effect is much longer (3 ns at $B = 0.43$ T) compared to the experimentally obtained T^*_2 values. Thus, we conclude that the observed spin-dephasing does not relate to the random or "fish scale" orientation of NPLs.

It is interesting that, even in the case of the randomly oriented ensemble, the precession frequency is found to be close to the transverse frequency Ω_x. For small magnetic fields, $g_{\text{eff}} = 1.86$, while for large fields, we find that $g_{\text{eff}} = 1.87$.

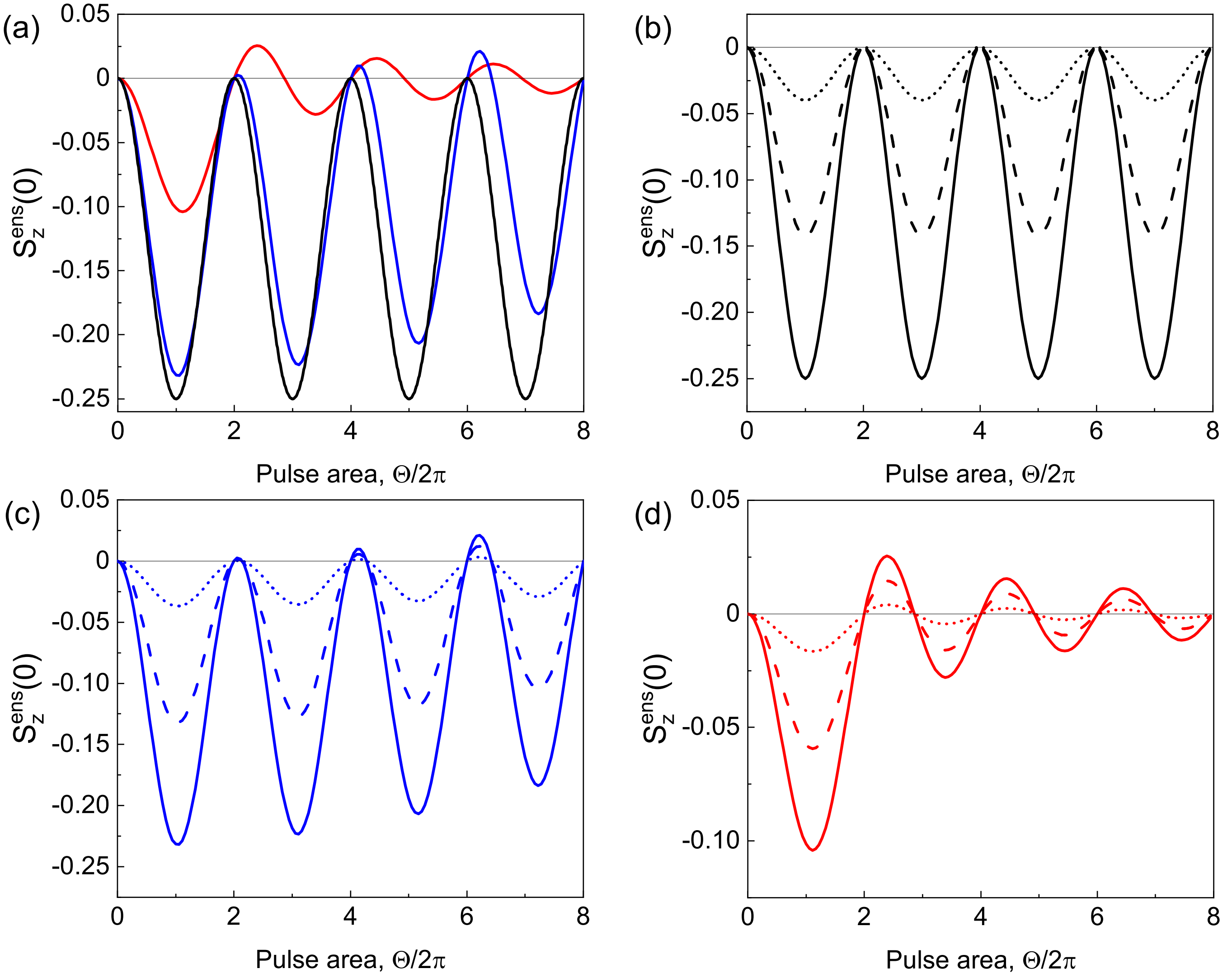

Figure S8. (a) Dependence of the initial spin polarization $S_z^{\text{ens}}(t=0)$ on the pump pulse area Θ for the bimodal ensemble (black), the "fish scale" ensemble (blue), and the randomly oriented ensemble (red) for zero detuning $\chi = 0$. (b-d) The same dependencies for three types of NPL ensembles with $\chi = 0$, 0.25, 0.5 shown by solid, dashed, and dotted lines, respectively.

This result is important because pioneer papers on TRFR in ensembles of wurtzite CdSe NCs claimed that, in the case of the randomly oriented ensemble, one should observe either two precession frequencies [S16] corresponding to longitudinal and transverse g-factors, or a single frequency corresponding to the average g-factor [S13].

The last (but not the least) feature of the randomly oriented ensemble of NPLs is a pronounced asymmetric precession signal with respect to the zero level. It is provided by a non-oscillating component. The amplitude of the non-oscillating component is rather large, 1/4 of the oscillating component amplitude. The reason for this non-oscillating component is that the dielectric polarization is proportional to the average value of the electron spin along the anisotropy axis, and not along the direction of propagation of the probe pulse. If the anisotropy axis is tilted from the direction of the pump and probe pulses, and the magnetic field is applied in the Voigt geometry, then the projection of the electron spin on the anisotropy axis has a constant contribution due to the presence of the longitudinal component of the magnetic field in the NPL frame. The non-oscillating TRFR component, observed in wurtzite CdSe NCs [S5], was associated with the frozen spin polarization of heavy holes. Our result shows that electrons could be the origin of the non-oscillating component in randomly oriented ensembles of NPLs or NCs with large splitting between heavy and light holes.

The presented theoretical consideration shows that, in the case of anisotropic electron g-factor, the spin precession frequency for the ensemble of CdSe NPL is determined mostly by the transverse component of the g-factor, regardless of the spatial orientation of NPLs in the ensemble. We also show that the dependence of the initialized spin polarization

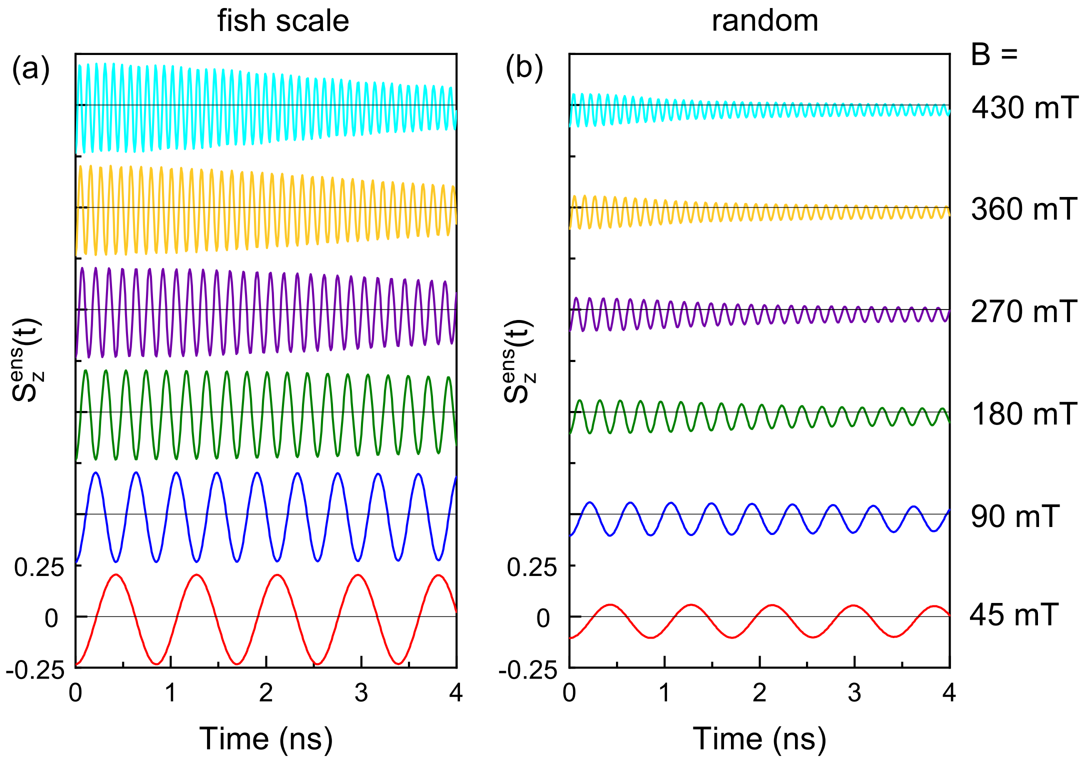

Figure S9. Time dependence of the $S_z^{\rm ens}$ for the "fish scale" ensemble (a) and the randomly oriented ensemble (b) for zero detuning of the pump pulse $\chi = 0$. The magnetic field used for the calculation equals (from the bottom to the top) 45, 90, 180, 270, 360, 430 mT.

on the area of the pump pulse can be used as a marker of the NPL orientation in the ensemble.

Tilted magnetic field

In the main text of the manuscript for the fitting of the TRFR signal in the tilted magnetic field, we used the following equation:

$$S(t) = S_0 \sin^2\alpha \cos(\omega_{\rm L} t) \exp(-t/T_2^*) + S_0 \cos^2\alpha \exp(-t/T_1). \tag{S21}$$

This equation suggests the isotropic electron g-factor. In the case of the anisotropic g-factor, one should use the following general equation:

$$S_z(t) = S_0 \frac{\Omega_x^2}{\Omega^2} \cos\Omega t \exp(-t/T_2^*) + S_0 \frac{\Omega_z^2}{\Omega^2} \exp(-t/T_1), \tag{S22}$$

with $\Omega_z = g_{||} \cos\alpha \mu_B B/\hbar$, $\Omega_x = g_\perp \sin\alpha \mu_B B/\hbar$, and $\Omega = \sqrt{\Omega_x^2 + \Omega_z^2}$. In Figure S11, we compare the dependencies for $S(t)$, calculated according to these two equations, using T_1 and T_2^* values determined in the main text. One can see a minor difference between the two calculated dependencies. This difference only affects the ratio between the oscillating and non-oscillating components, which we do not discuss. Thus, Equation (4) in the main text provides an accurate estimation of the T_1 and T_2^* times for the studied CdSe NPLs.

[S1] Feng, D. H.; Yakovlev, D. R.; Dubertret, B.; Bayer, M. Charge separation dynamics in CdSe/CdS core/shell nanoplatelets addressed by coherent electron spin precession, *ACS Nano* **2020**, *14*, 7237–7244.

[S2] Cragg, G. E.; Efros, A. L. Suppression of Auger processes in confined structures. *Nano Lett.* **2010**, *10*, 313–317.

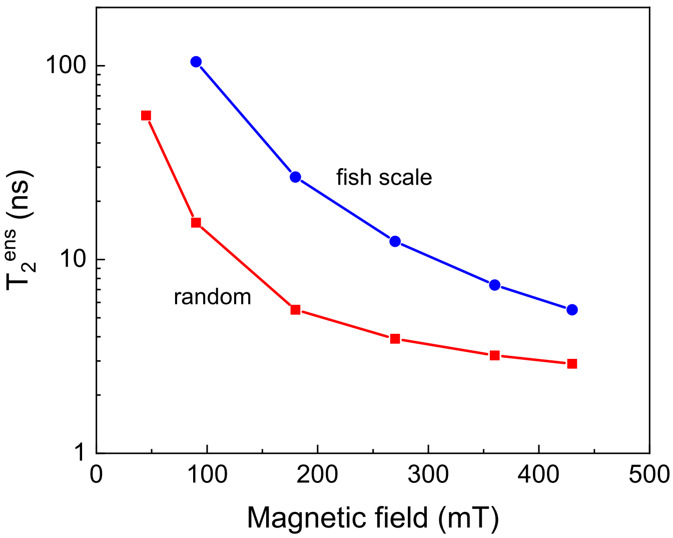

Figure S10. The magnetic field dependence of the spin-dephasing time related to the dispersion of the electron g-factor in the ensemble with "fish scale" stacking (blue) and the randomly oriented ensemble of NPL (red). The time T_2^{ens} is determined from the fitting of dependencies presented in Figure S9 by function $A_{\mathrm{nosc}} + A_{\mathrm{osc}} \cos(g_{\mathrm{eff}}\mu_B B/\hbar t)\exp(-t/T_2^{\mathrm{ens}})$.

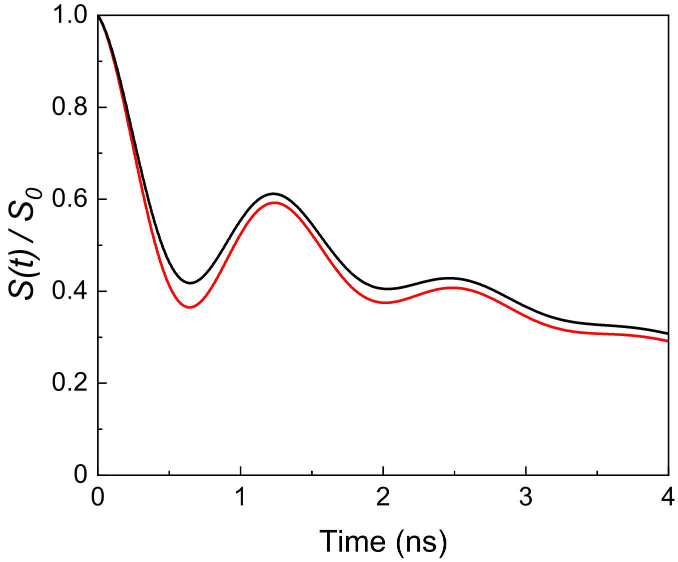

Figure S11. Calculated time dependence of the spin polarizations $S(t) = S_0(\Omega_z^2/\Omega^2 \exp(-t/T_1) + \Omega_x^2/\Omega^2 \cos \Omega t \exp(-t/T_2))$ with $\Omega_z = g_{||} \cos \alpha \mu_B B/\hbar$, $\Omega_x = g_\perp \sin \alpha \mu_B B/\hbar$ for $B = 50$ mT, and $\alpha = 35°$. The black line is calculated assuming an isotropic g-factor $g_\perp = g_{||} = 1.88$. The red line is calculated with $g_\perp = 1.88$ and $g_{||} = 1.73$. Relaxation times $T_2^* = 1$ ns and $T_1 = 5$ ns are used.

[S3] Shornikova, E. V.; Yakovlev, D. R.; Biadala, L; Crooker, S. A.; Belykh, V. V.; Kochiev, M. V.; Kuntzmann, A.; Nasilowski, M.; Dubertet, B.; Bayer, M. Negatively charged excitons in CdSe nanoplatelets. *Nano Lett.* **2020**, *20*, 1370–1377.

[S4] Semina, M. A.; Golovatenko, A. A.; Rodina, A. V. Influence of the spin-orbit split-off valence band on the hole g-factor in semiconductor nanocrystals. *Phys. Rev. B* **2021**, *104*, 205423.

[S5] Qiang, G.; Zhukov, E. A.; Evers, E.; Yakovlev, D. R.; Golovatenko, A. A.; Rodina, A. V.; Onushchenko, A. A.; Bayer, M.
Electron spin coherence in CdSe nanocrystals in a glass matrix. *ACS Nano* **2022**, *16*, 18838–18848.

[S6] Yugova, I. A.; Glazov, M. M.; Ivchenko, E. L.; Efros, Al. L. Pump-probe Faraday rotation and ellipticity in an ensemble of singly charged quantum dots, *Phys. Rev. B* **2009**, *80*, 104436.

[S7] Glazov, M. M. Coherent spin dynamics of electrons and excitons in nanostructures (a review). *Phys. Sol. State* **2012**, *54*, 1–27.

[S8] Smirnov D. S., Glazov, M. M. Spin coherence generation and detection in spherical nanocrystals, *J. Phys.: Condens.*

Matter **2012**, *24*, 345302.

[S9] Shornikova, E. V.; Yakovlev, D. R.; Gippius, N. N.; Qiang, G.; Dubertet, B.; Khan, A. H.; Giacomo, A. D.; Moreels, I.; Bayer, M. Exciton binding energy in CdSe nanoplatelets measured by one- and two-photon absorption. *Nano Lett.* **2021**, *21*, 10525–10531.

[S10] Shornikova, E. V.; Biadala, L.; Yakovlev, D. R.; Feng, D. H.; Sapega, V. F.; Flipo, N.; Golovatenko, A. A.; Semina, M. A.; Rodina, A. V.; Mitioglu, A. A.; Ballottin, M. V.; Christianen, P. C. M.; Kusrayev, Yu. G.; Nasilowski M.; Dubertret, B.; Bayer, M. Electron and hole g-factors and spin dynamics of negatively charged excitons in CdSe/CdS colloidal nanoplatelets with thick shells, *Nano Lett.* **2018**, *18*, 373–380.

[S11] Kudlacik, D.; Sapega, V. F.; Yakovlev, D. R.; Kalitukha, I. V.; Shornikova, E. V.; Rodina, A. V.; Ivchenko, E. L.; Dimitriev, G. S.; Nasilowski, M.; Dubertet, B.; Bayer, M. Single and double electron spin-flip Raman scattering in CdSe colloidal nanoplatelets. *Nano Lett.* **2020**, *20*, 517–525.

[S12] Gupta, J. A.; Awschalom, D. D.; Efros, Al. L.; Rodina, A. V. Spin dynamics in semiconductor nanocrystals. *Phys. Rev. B* **2002**, *66*, 125307.

[S13] Stern, N. P.; Poggio, M.; Bartl, M. H., Hu, E. L., Stucky, G. D., Awschalom, D. D. Spin dynamics in electrochemically charged CdSe quantum dots. *Phys. Rev. B* **2005**,*72*, 161303(R).

[S14] Kirstein, E.; Kopteva, N. E.; Yakovlev, D. R.; Zhukov, E. A.; Kolobkova, E. V.; Kuznetsova, M. S.; Belykh, V. V.; Yugova, I. A.; Glazov, M. M.; Bayer, M.; Greilich, A. Mode locking of hole spin coherences in $CsPb(Cl,Br)_3$ perovskite nanocrystals. *Nature Commun.* **2023**, *14*, 699.

[S15] Rosen, N.; Zener, C. Double Stern-Gerlach experiment and related collision phenomena. *Phys. Rev.*, **1932**, *40*, 502.

[S16] Chen, P.; Whaley, K. B. Magneto-optical response of CdSe nanostructures. *Phys. Rev. B* **2003**, *70*, 045311.
